# TDP-43 dysfunction results in R-loop accumulation and DNA replication defects

**DOI:** 10.1242/jcs.244129

**Published:** 2020-10-30

**Authors:** Matthew Wood, Annabel Quinet, Yea-Lih Lin, Albert A. Davis, Philippe Pasero, Yuna M. Ayala, Alessandro Vindigni

**Affiliations:** 1 Division of Oncology, Department of Medicine, Washington University in St. Louis, St. Louis, MO 63110, USA; 2 Edward A. Doisy Department of Biochemistry and Molecular Biology, Saint Louis University School of Medicine, St. Louis, MO 63104, USA; 3 Institut de Génétique Humaine, CNRS et Université de Montpellier, Equipe labélisée Ligue contre le Cancer, Montpellier 34396, France; 4 Department of Neurology, Washington University in St. Louis, St. Louis, MO 63110, USA

**Keywords:** DNA Replication, R-loops, RNA:DNA hybrids, TDP-43, TARDBP

## Abstract

TAR DNA-binding protein 43 (TDP-43; also known as TARDBP) is an RNA-binding protein whose aggregation is a hallmark of the neurodegenerative disorders amyotrophic lateral sclerosis and frontotemporal dementia. TDP-43 loss increases DNA damage and compromises cell viability, but the actual function of TDP-43 in preventing genome instability remains unclear. Here, we show that loss of TDP-43 increases R-loop formation in a transcription-dependent manner and results in DNA replication stress. TDP-43 nucleic-acid-binding and self-assembly activities are important in inhibiting R-loop accumulation and preserving normal DNA replication. We also found that TDP-43 cytoplasmic aggregation impairs TDP-43 function in R-loop regulation. Furthermore, increased R-loop accumulation and DNA damage is observed in neurons upon loss of TDP-43. Together, our findings indicate that TDP-43 function and normal protein homeostasis are crucial in maintaining genomic stability through a co-transcriptional process that prevents aberrant R-loop accumulation. We propose that the increased R-loop formation and genomic instability associated with TDP-43 loss are linked to the pathogenesis of TDP-43 proteinopathies.

This article has an associated First Person interview with the first author of the paper.

## Abstract (precis)

**Summary:** TDP-43 deficiency increases the transcription-dependent accumulation of R-loops and perturbs DNA replication in proliferating cells. This R-loop accumulation, in turn, compromises genomic integrity in human cells, including neurons.

## INTRODUCTION

R-loops are three-stranded nucleic acid structures that form during transcription when RNA annealing to the template DNA strand displaces the complementary DNA strand. Although R-loops play important physiological roles in several cellular processes, aberrant accumulation of R-loop structures can hamper DNA replication, repair and transcription and lead to genomic instability (Gan et al., 2011; Groh and Gromak, 2014; Huertas and Aguilera, 2003; Li and Manley, 2005; Tuduri et al., 2009; Wahba et al., 2011).

Increased DNA damage linked to R-loop accumulation was recently proposed to play a role in the neurotoxicity of two overlapping neurodegenerative disorders, amyotrophic lateral sclerosis (ALS) and frontotemporal dementia (FTD) (Walker et al., 2017). These disorders are connected to the aggregation and dysfunction of RNA-binding proteins, including proteins that regulate R-loops, such as senataxin (SETX) and ataxin-2 (also known as ATXN2) (Richard and Manley, 2017). In addition, the most common genetic link to familial and sporadic ALS and FTD is an intronic hexanucleotide repeat expansion (G_4_C_2_) in the *C9Orf72* gene (C9-HRE), leading to increased R-loop formation and genomic instability in patient spinal cord tissue and animal models (Abu Diab et al., 2018; Haeusler et al., 2014; Walker et al., 2017). This collective evidence suggests that DNA damage and aberrant R-loop accumulation play a previously unappreciated role in ALS and FTD pathogenesis.

TAR DNA-binding protein (TDP-43; also known as TARDBP) is an RNA- and DNA-binding protein whose aggregation is a hallmark of ALS and characterizes approximately half of FTD cases (Arai et al., 2006; Neumann et al., 2006). Furthermore, the aggregates are a secondary pathology in a wide spectrum of neurodegenerative disorders, including occurrence in 50% of patients with Alzheimer's disease (Amador-Ortiz et al., 2007; Josephs et al., 2008; Nelson et al., 2019; Wilson et al., 2013). Dominant mutations in the TDP-43 gene are causative of approximately 4% of familial ALS and 1% of sporadic ALS (Lagier-Tourenne et al., 2010), strongly suggesting that TDP-43 dysfunction is linked to neurodegeneration. TDP-43 inclusions coincide with a dramatic reduction in normal nuclear TDP-43 localization, implying a loss of function upon aggregate accumulation (Neumann et al., 2006). This is viewed as a major factor impeding neuronal function, as TDP-43 is essential for development and survival in animal models and cell-based studies (Ayala et al., 2008a; Chiang et al., 2010; Feiguin et al., 2009; Schmid et al., 2013; Sephton et al., 2010). Whether disease results from aggregate toxicity, from sequestration of TDP-43 into aggregates and the consequent loss of protein function (Neumann et al., 2006), or from a combination of both mechanisms is unclear.

TDP-43 is a highly conserved heterogeneous nuclear ribonucleoprotein regulating RNA processing and controlling the expression of hundreds of genes through direct binding to RNA (Polymenidou et al., 2011; Rot et al., 2017; Tollervey et al., 2011). The most well-established TDP-43 function is regulation of splicing and alternative polyadenylation (Ayala et al., 2006; Buratti et al., 2001; Ling et al., 2015; Mercado et al., 2005; Polymenidou et al., 2011; Rot et al., 2017; Tollervey et al., 2011). TDP-43 is organized into multiple folded domains and a predominantly disordered C-terminal region. The TDP-43 N-terminal domain mediates oligomerization (Afroz et al., 2017; Mompeán et al., 2017; Wang et al., 2018; Zhang et al., 2013) and is followed by two canonical RNA-binding motifs (RRMs), of which RRM1 is necessary and sufficient to bind RNA (Buratti and Baralle, 2001). The C-terminal tail is a prion-like or low complexity sequence domain (LCD) that mediates protein interactions (Ayala et al., 2011; D'Ambrogio et al., 2009) and is also a strong driver of aggregation (Fuentealba et al., 2010).

In human cells, including motor neurons, TDP-43 loss increases DNA damage and compromises cell viability (Ayala et al., 2008a; Mitra et al., 2019). However, TDP-43 has no reported roles in DNA repair and the molecular basis for the increased DNA damage associated with TDP-43 loss remains unclear. Interestingly, a recent study showed that this damage can be partially rescued upon overexpression of RNase H1, an enzyme involved in R-loop cleavage, suggesting that there might be a molecular link between the increased DNA damage observed upon TDP-43 loss and R-loop accumulation (Hill et al., 2016). Here, we provide the first evidence that TDP-43 regulates R-loop accumulation and that this novel function of TDP-43 is crucial in preventing replication fork perturbations and DNA damage. Moreover, we show that R-loop accumulation associated with TDP-43 dysfunction is transcription dependent. We also find that both the RNA-binding and oligomerization functions of TDP-43 are required to prevent R-loop formation and promote normal replication fork progression. Collectively, our work uncovers a previously unappreciated function of TDP-43 in the maintenance of genomic integrity and cell viability, providing new insights into the pathogenesis of TDP-43 proteinopathies.

## RESULTS

### Loss of TDP-43 leads to DNA breaks and impairs replication fork progression

**Fig. 1.**
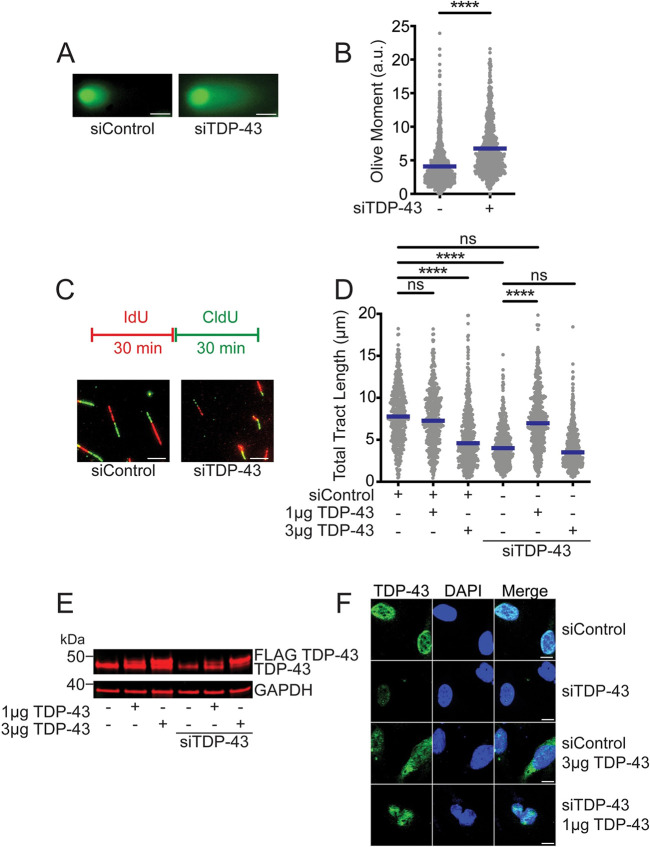
**TDP-43 knockdown induces DNA breaks and replication stress.** (A) Neutral comet assay detecting DSBs in HeLa cells treated with control siRNA (siControl) or siRNA targeting TDP-43 (siTDP-43). Representative images of comets are shown. (B) Quantification of comet Olive moment. Data are represented as dot plot and means from five repeats, with mean values. *n*≥100 comets scored for each data set. ****P<0.0001 (unpaired *t*-test). (C) Schematic of the single-molecule DNA fiber tract labeling. HeLa cells transfected with siControl or siTDP-43 were sequentially labeled with IdU and CldU for 30 min each. Representative DNA fiber images after transfection with siControl or siTDP-43 are shown. (D) Size distribution of total tract length (IdU+CldU) in HeLa cells after transfection with control siRNA or siTDP-43 and siRNA-resistant WT FLAG-TDP-43 construct. HeLa cells treated with siControl or siTDP-43 were transfected with either 1 or 3 µg of WT FLAG-TDP43 vector. Data are pooled from three independent experiments and shown as dot plot. Bars represent the median of *n*≥150 tracts scored for each data set. ****P<0.0001; ns, non-significant (Kruskal–Wallis test with Dunn's multiple comparisons test). (E) Protein expression upon TDP-43 depletion (siTDP-43) and transfection with siRNA-resistant WT FLAG-TDP-43 construct. A representative western blot from three independent experiments is shown. (F) Representative immunofluorescence images of TDP-43 cellular localization after TDP-43 depletion and siRNA-resistant WT FLAG-TDP-43 vector expression. Scale bars: 5 µm (A,C), 10 µm (F).

TDP-43 plays a mechanistically ill-defined function in preventing DNA breaks and genomic instability (Ayala et al., 2008a; Hill et al., 2016; Mitra et al., 2019). In agreement with previous findings (Ayala et al., 2008a), we found that loss of TDP-43 by siRNA-mediated knockdown (KD) in human HeLa cells led to increased levels of phosphorylated histone H2AX (Ser139, γH2AX), compared with TDP-43-proficient controls (Fig. S1A). This increase in γH2AX levels was accompanied by an increase in the levels of double-stranded DNA breaks (DSBs), as detected by neutral comet assay. Transfection with siRNA targeting TDP-43 (siTDP-43) caused a 1.6-fold increase in the average Olive tail moment measured by comet assays relative to cells treated with non-targeting control siRNA (siControl; Fig. 1A,B). Accumulation of DSBs upon loss of TDP-43 was in turn associated with increased accumulation of cells in the G2/M phase of the cell cycle (Fig. S1B,C), consistent with a previous report (Ayala et al., 2008a). To differentiate G2 phase from mitosis, we compared the levels of the mitotic marker phospho-histone H3 (Ser10) (Lee et al., 2014) in control versus TDP-43 KD cells by immunoblot. In contrast to cells treated with control siRNA, the levels of phospho-histone H3 were not increased in TDP-43 KD cells upon induction of mitotic arrest by nocodazole treatment (Fig. S1D), suggesting that TDP-43 loss activates a checkpoint-mediated arrest, preventing cell progression into mitosis. Moreover, TDP-43 KD led to a defect in the incorporation of the thymidine analog 5-ethynyl-2′-deoxyuridine (EdU) during S phase (Fig. S1B), suggesting that TDP-43 loss perturbs DNA replication and promotes DNA damage accumulation, activating a G2 phase cell cycle arrest.

To further investigate the replication defects associated with TDP-43 loss, we measured replication fork progression in TDP-43 KD cells by genome-wide single molecule DNA fiber analysis (Quinet et al., 2017). HeLa cells were labeled with 5-iodo-2′-deoxyuridine (IdU) for 30 min and subsequently with 5-chloro-2′-deoxyuridine (CldU) for an additional 30 min (Fig. 1C). Loss of TDP-43 resulted in significant shortening of DNA replication tracts compared with control cells, suggesting that disruption of TDP-43 function perturbs replication fork progression, leading to fork slowing (Fig. 1D, lanes 1 and 4). Individual IdU and CldU tract lengths mirrored the shortening phenotypes seen by measuring the total tract length (Fig. S2A,B). Accordingly, the average ratio of IdU- and CldU-labeled tract lengths was close to one in both control and TDP-43 KD cells, indicating that both tracts were equally perturbed after TDP-43 loss (Fig. S2C). Complementation experiments using 1 µg of siRNA-resistant, fully functional TDP-43 (WT) in TDP-43 KD cells showed complete rescue of replication fork length (Fig. 1D,E, lane 5), confirming that the replication tract shortening phenotype was specifically caused by the loss of TDP-43. However, we did not observe the same rescue phenotype when we transfected the TDP-43 KD cells with a higher amount of the siRNA-resistant TDP-43 WT vector (Fig. 1D, lane 6). Moreover, transfection of control cells with 1 µg WT-TDP-43 vector did not significantly affect DNA fiber tract length (Fig. 1D, lane 2), whereas transfection with higher levels of TDP-43 (3 µg) led to nearly the same extent of tract shortening observed upon loss of TDP-43 (Fig. 1D, lane 3). At the same time, our immunofluorescence analysis showed that TDP-43 is primarily localized in the nuclei when control cells are transfected with 1 µg WT-TDP-43 vector, whereas transfection with 3 μg of the vector resulted in high levels of protein overexpression leading to TDP-43 mislocalization, as seen by increased levels in the cytoplasm and concomitant nuclear loss (Fig. 1F). This phenotype resembles pathological features in TDP-43 proteinopathies associated with TDP-43 aggregation and cell toxicity (Neumann et al., 2006). Interestingly, mislocalization of TDP-43 after overexpression strongly correlated with the presence of the DNA damage marker γH2AX (Fig. S2D). Hence, our results suggest that TDP-43 mislocalization and aggregation also impair DNA replication through a loss of nuclear function mechanism.

**Fig. 2.**
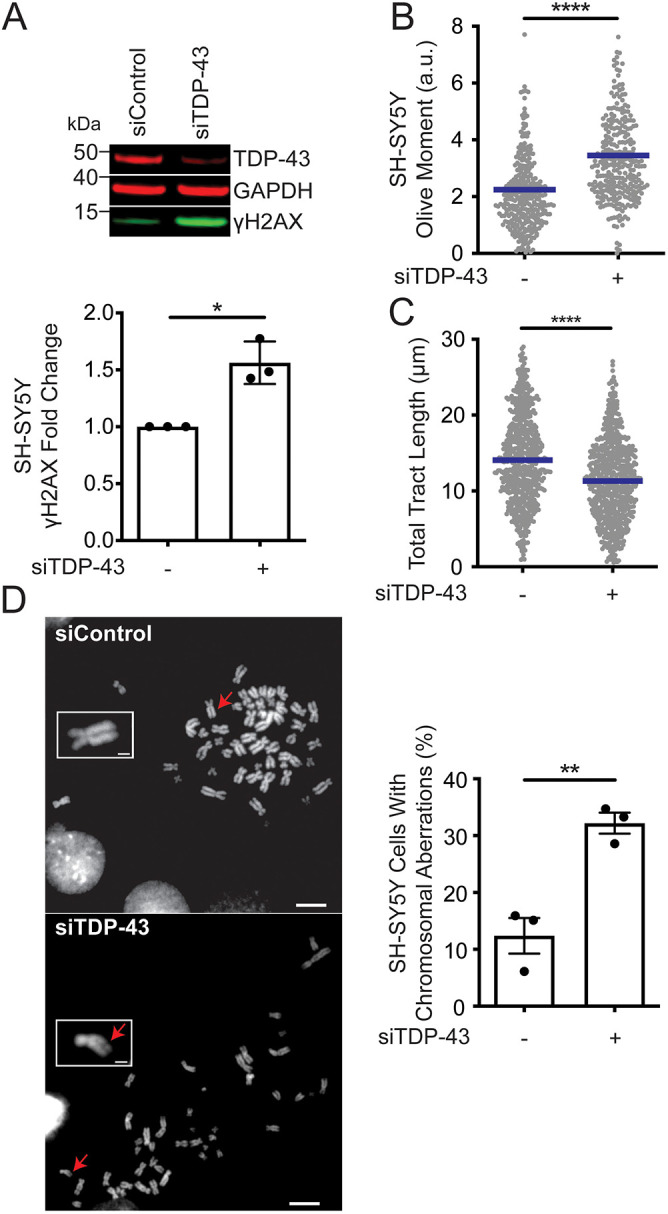
**TDP-43 knockdown induces genomic instability in neuroblastoma cells.** (A) TDP-43 protein expression and the DNA damage marker γH2AX after knockdown with siTDP-43 in SH-SY5Y cells. Representative western blot (top) and quantification of γH2AX expression levels (bottom) after TDP-43 knockdown in SH-SY5Y cells. Data show fold increase in γH2AX expression compared with siControl-treated samples, from three independent western blots (mean±s.e.m.). *P=0.035 (paired *t*-test). (B) Quantification of Olive moments detected by neutral comet assay represented as a dot plot. Bars represent the mean of a total of five repeats; *n*≥100 comets scored for each data set. ****P<0.0001 (unpaired *t*-test). (C) Size distribution of total tract length (IdU+CldU) in SH-SY5Y cells after transfection with siControl or siTDP-43. Data are pooled from three independent experiments and shown as dot plot. Bars represent the median of *n*≥150 tracts scored for each data set. ****P<0.0001 (unpaired *t*-test). (D) Accumulation of chromosomal aberrations detected upon chromosome spread. Left: Representative images of chromosomes after SH-SY5Y transfection with siControl or siTDP-43. Red arrows point to the chromosome shown in the insert. A chromosomal break is highlighted in siTDP-43. Scale bars: 1 µm. Right: Percentage of cells with chromosomal abnormalities per data set (mean±s.e.m.); *n*≥50 metaphases scored for each of the three individual data sets. **P=0.0054 (paired *t*-test).

Next, we tested whether loss of TDP-43 disrupts DNA replication function and causes genomic instability in neuronal-like cells. Human SH-SY5Y cells showed a 1.5-fold increase in γH2AX levels compared with controls, as well as an increase in DSBs (measured by Olive tail moment) upon TDP-43 KD (Fig. 2A,B). We also observed a statistically significant reduction in DNA fiber tract length in the SH-SY5Y TDP-43 KD cells compared with controls (Fig. 2C), although this effect was less marked than in HeLa cells (Fig. 1C, lanes 1 and 4). Moreover, we observed a significant threefold increase in chromosomal aberration in TDP-43 KD cells relative to control cells (Fig. 2D). Collectively, these results suggest that TDP-43 plays a major role in preserving genomic stability and DNA replication function in different human cell lines.

### TDP-43 loss leads to R-loop accumulation

**Fig. 3.**
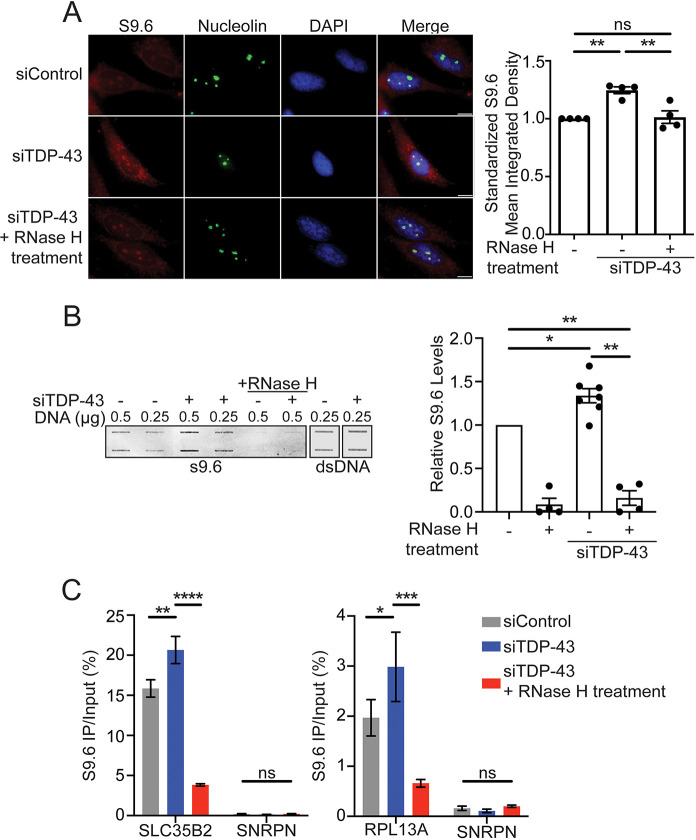
**TDP-43 loss increases accumulation of R-loops.** (A) Left: Representative immunofluorescence images of HeLa cells depleted for TDP-43 (siTDP-43) and treated with the RNase H nuclease probed with R-loop-specific (S9.6) and nucleolin antibodies. Right: Quantification of nuclear S9.6 intensity signal, minus nucleolar signal, shown as relative to siRNA control and untreated samples. *n*≥100 cells scored for each data set. Data are represented as mean±s.e.m. from four independent experiments. **P=0.0097; ns, non-significant (paired *t*-test). (B) Slot blot analysis of siRNA-treated HeLa nuclear fractions. Left: Representative image of 0.25 or 0.5 μg DNA loaded in duplicate and probed with S9.6. Samples (0.5 μg) were also treated with RNase H prior to loading. DNA (0.25 μg) was probed with dsDNA-specific antibody as loading control. The blot was probed with R-loop (S9.6) or dsDNA-specific antibodies. Right: Quantification of S9.6 signal in siTDP-43-treated samples in the presence and absence of RNase H relative to siControl using LI-COR Image Studio. Values show the S9.6 enrichment from 0.25 and 0.5 μg DNA loadings combined (*n*=7, mean±s.e.m.). *P=0.02, **P≤0.01 (mixed-effects statistical analysis with Geisser-Greenhouse correction; Tukey's multiple comparisons tests with individual variances computed for each comparison). (C) DRIP-qPCR analysis of HeLa cells treated with siControl or siTDP-43 with and without RNase H1 treatment. Left: The R-loop prone sequence SLC35B2 and an additional known negative control sequence *SNRPN* were probed. Data are represented as mean±s.e.m. from two independent experiments. **P=0.0062, ****P<0.0001; ns, non-significant (left) (two-way ANOVA with Holm-Sidak's multiple comparisons test, with a single pooled variance). Right: The R-loop prone sequence *RPL13A* and an additional known negative control sequence *SNRPN* were probed. Data are represented as mean±s.e.m. from three independent experiments. *P=0.0451, ***P=0.0008; ns, non-significant (two-way ANOVA with Holm-Sidak's multiple comparisons test, with a single pooled variance).

Replication fork slowing may be caused by obstructions present on DNA, such as DNA breaks and the presence of RNA:DNA hybrid structures (reviewed by Gómez-González and Aguilera, 2019) (Gan et al., 2011; Huertas and Aguilera, 2003; Tuduri et al., 2009; Wahba et al., 2011). Because of the previously established function of TDP-43 in RNA metabolism, we asked whether the replication defects observed upon TDP-43 downregulation were caused by increased accumulation of R-loops. We quantified nuclear R-loop formation by immunofluorescence using the S9.6 antibody (Boguslawski et al., 1986) in control and TDP-43 KD HeLa cells, as previously described (Sollier et al., 2014) (Fig. 3A). Consistent with results reported by other labs, S9.6 staining was observed both in the nucleus as well as the cytoplasm, also colocalizing with nucleoli (García-Rubio et al., 2015; Sollier et al., 2014). We only measured nuclear S9.6 staining and subtracted nucleolar signal according to previously established methodologies for scoring R-loop levels associated with nuclear function (Aguilera and García-Muse, 2012; Koo et al., 2015; Sollier et al., 2014; Xu and Clayton, 1996). Importantly, loss of TDP-43 did not change nuceolin expression levels, showing that loss of TDP-43 does not significantly affect nucleolar detection (Fig. S3A). We found that TDP-43 KD consistently and significantly increased S9.6 levels compared with control-treated cells (Fig. 3A). To confirm that the signal observed upon staining with the S9.6 antibody was due to the accumulation of R-loops, we repeated the experiment following RNase H endonuclease treatment to specifically degrade RNA:DNA hybrids in TDP-43 KD cells (Cerritelli and Crouch, 2009; Wahba et al., 2011). RNase H1 treatment significantly decreased S9.6 levels in the nucleus of TDP-43 KD cells (Fig. 3A), supporting the idea that TDP-43 loss leads to an accumulation of R-loops. We further confirmed our observations, measuring R-loop levels by slot blot analysis in isolated nuclei from HeLa cells that were treated with control siRNA or siRNA targeting TDP-43 (Fig. 3B). Extraction of the nuclear compartment, where TDP-43 is predominantly found (Ayala et al., 2008b), allowed us to remove mitochondrial R-loop accumulation (Fig. S3B). Probing with S9.6 showed a 40% increase in R-loop levels in TDP-43 KD cells relative to control. The S9.6 signal was sensitive to RNase H treatment, confirming that most of this signal corresponds to nuclear R-loop structures. In parallel, we performed DNA-RNA immunoprecipitation combined with quantitative PCR (DRIP-qPCR) using S9.6 to quantify RNA:DNA hybrids at different gene loci enriched in R-loops (Fig. 3C) (Promonet et al., 2020). Our data further confirmed that depletion of TDP-43 promoted the enrichment of R-loops at these genes (*SLC35B2* and *RPL13A*). As negative control, we probed *SNRPN*, which does not accumulate R-loops (Bhatia et al., 2014; Herrera-Moyano et al., 2014; Promonet et al., 2020), and observed no significant increase upon TDP-43 downregulation. RNase H1 treatment significantly reduced the DRIP signal in TDP-43 KD cells, further confirming the specificity of R-loops detection using this approach.

**Fig. 4.**
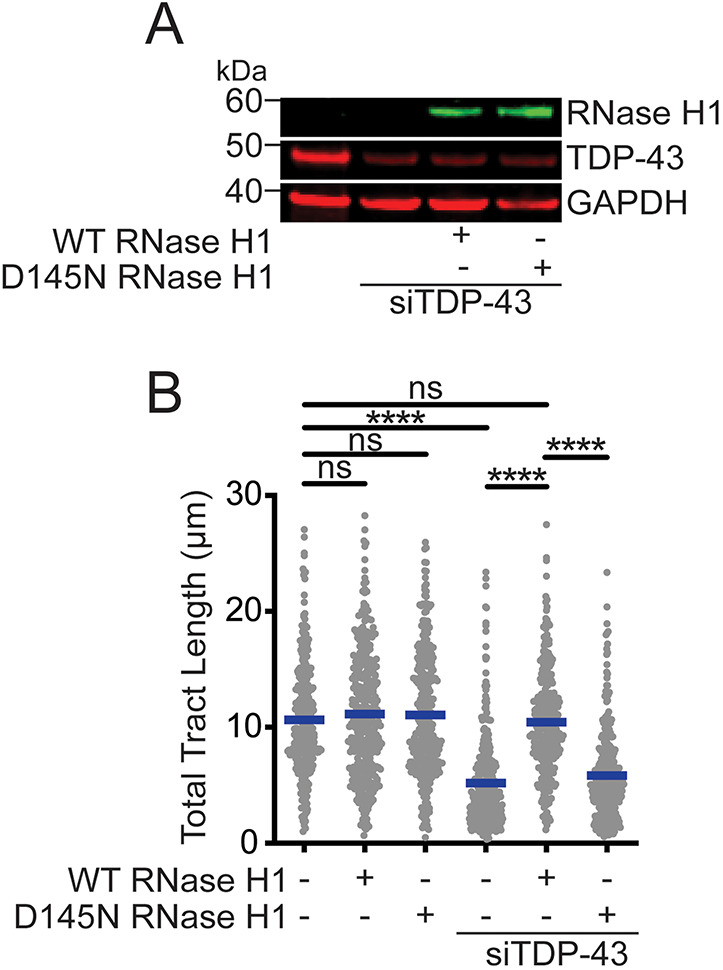
**R-loop resolution by RNase H1 resolves replication stress in TDP-43 depleted cells.** (A) Protein expression of TDP-43 and RNase H1 after TDP-43 knockdown and transfection with wild-type (WT) or nuclease mutant D145N RNase H1 constructs. A representative western blot from three experiments is shown. (B) Size distribution of total tract length (IdU+CldU) in HeLa cells after transfection with siControl or siTDP-43 and WT or nuclease mutant D145N RNase H1 constructs. Data are pooled from three independent experiments and shown as dot plot. Bars represent the median of *n*≥150 tracts scored for each data set. ****P<0.0001; ns, non-significant (Kruskal–Wallis test with Dunn's multiple comparisons test).

To test the contribution of R-loop accumulation to replication stress after TDP-43 downregulation, we measured DNA tract shortening upon RNase H1 overexpression (Fig. 4A). Overexpression of RNase H1 in cells completely rescued the DNA tract shortening phenotype associated with loss of TDP-43 (Fig. 4B, lane 5). On the other hand, overexpression of the catalytically dead RNase H1 mutant D145N (Wu et al., 2001) failed to rescue DNA tract shortening in TDP-43 KD cells (Fig. 4B, lane 6). As a control, overexpression of either WT or D145N RNase H1 did not alter replication tract length in RNAi control treated cells (Fig. 4B, lanes 2 and 3). Together, our results strongly suggest that TDP-43 loss increases the accumulation of R-loops and that this accumulation disrupts replication fork progression.

### R-loop accumulation upon TDP-43 downregulation is transcription dependent

**Fig. 5.**
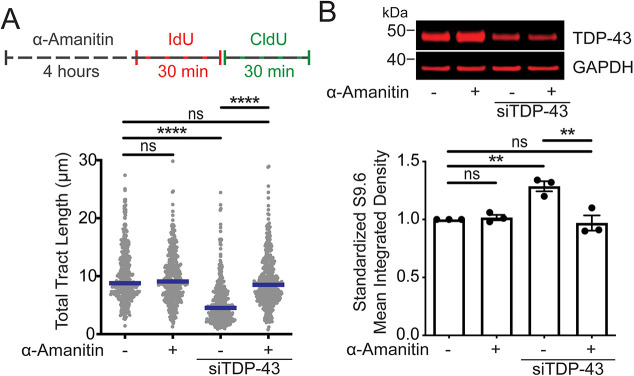
**TDP-43-mediated replication stress is dependent on transcription.** (A) Top: Schematic of the DNA fiber assay with the transcription inhibitor α-amanitin. α-Amanitin (5 µM) was added to the cell medium for 4 h prior to the DNA fiber assay and was kept in the medium throughout labeling with IdU and CldU thymidine analogs. Bottom: Size distribution of total tract length (IdU+CldU) in HeLa cells transfected with siControl or siTDP-43 and treated with α-amanitin. Data are pooled from three independent experiments and are shown as dot plot. Bars represent the median of *n*≥150 tracts scored for each data set. ****P<0.0001; ns, non-significant (Kruskal–Wallis test with Dunn's multiple comparisons test). (B) Top: TDP-43 protein expression after knockdown with siTDP-43 and α-amanitin treatment as detected by western blot. Bottom: Quantification of S9.6 intensity signal relative to siControl, untreated samples. *n*≥100 cells scored for each data set. Data are represented as mean±s.e.m. from five independent experiments. **P≤0.0088; ns, non-significant (repeated measure one-way ANOVA with Tukey's multiple comparisons test, with a single pooled variance).

RNA-binding proteins have been previously shown to prevent R-loop formation during transcription by coating the nascent RNA and inhibiting annealing to the DNA template. This process was first characterized for the serine/arginine-rich splicing factor 1 (SRSF1, also known as ASF/SF2), resulting in a model whereby SRSF1 binds G-rich nascent RNA and prevents RNA:DNA hybrid formation (Li and Manley, 2005). Loss of SRSF1 increases replication stress, DNA breaks, chromosomal aberrations and apoptosis (Tuduri et al., 2009). We asked whether TDP-43 regulation of R-loop accumulation is also a co-transcriptional process by inhibiting RNA Pol II in control and TDP-43 KD cells. To this end, we treated control and TDP-43 KD Hela cells with α-amanitin (Gong et al., 2004) for 4 h prior to and during nucleotide labeling for DNA fiber analysis (Fig. 5A). The 4 h treatment ensures that transcription is fully stopped and that all previously formed R-loops have been fully removed (Sanz et al., 2016). We found that treatment with α-amanitin rescued the replication tract shortening phenotype of TDP-43 KD cells (Fig. 5A, lanes 3 and 4). Moreover, α-amanitin treatment decreased R-loop levels in TDP-43 KD cells, as assessed by S9.6 staining, preventing the increase of R-loops as a result of TDP-43 downregulation (Fig. 5B). Importantly, treatment with α-amanitin did not alter TDP-43 protein expression levels (Fig. S4A). We obtained similar results by inhibiting RNA Pol II transcription through an alternative mechanism using actinomycin D (Act D) (Fig. S4B,C). DNA fiber analysis was carried out with control and TDP-43 KD cells treated with Act D for 6 h prior to DNA fiber labeling. As observed with α-amanitin, the total DNA tract length in TDP-43 KD was almost completely restored in the presence of Act D, compared with controls (Fig. S4B, lanes 3 and 4). Moreover, the levels of R-loop accumulation were not significantly different between control and TDP-43 KD cells treated with Act D (Fig. S4C). These results suggest that R-loop accumulation and replication fork slowing upon TDP-43 downregulation depend on transcription. Similar to the model proposed for SRSF1, we speculate that TDP-43 associates with nascent RNA to prevent annealing to template DNA, which would otherwise block replication fork progression.

### The RNA binding and oligomerization functions of TDP-43 are required for R-loop regulation

**Fig. 6.**
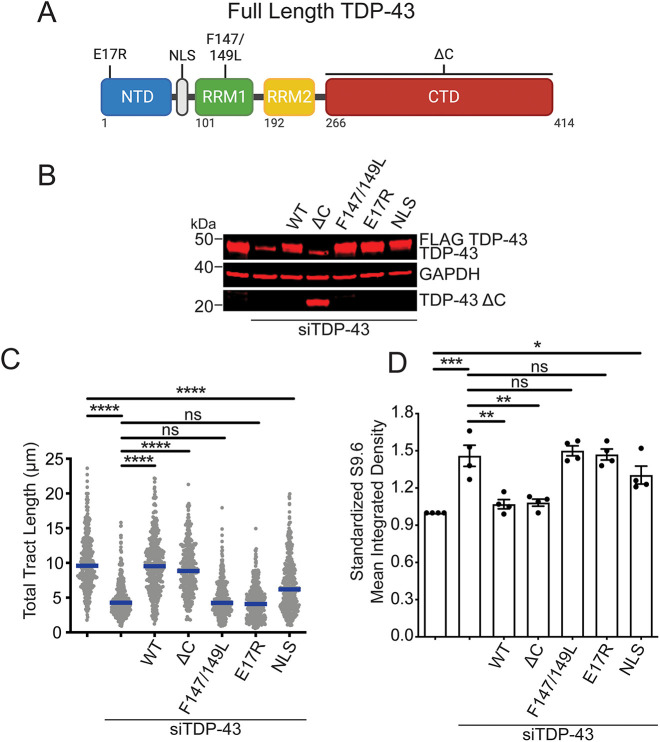
**Crucial TDP-43 domains in R-loop regulation.** (A) Schematic of TDP-43 domains: N-terminal domain (NTD), nuclear localization sequence (NLS), RNA recognition motifs (RRM1 and RRM2) and C-terminal domain (CTD). The approximate positions of applicable mutations are indicated. (B) TDP-43 protein expression upon TDP-43 depletion (siTDP-43) and transfection of the siRNA-resistant TDP-43 mutants shown in A. Representative western blots from three independent experiments are shown. (C) Size distribution of total tract length (IdU+CldU) in HeLa cells after transfection with siControl or siTDP-43 and the siRNA resistant TDP-43 mutants. Data are pooled from three independent experiments and shown as dot plot. Bars represent the median of *n*≥150 tracts scored for each data set. ****P<0.0001 (Kruskal–Wallis test with Dunn's multiple comparisons test). (D) Quantification of S9.6 intensity signal in HeLa cells transfected with siTDP-43 and the siRNA-resistant TDP-43 mutants. *n*≥100 cells scored for each data set. Data are represented as mean±s.e.m. relative to control cells from five independent experiments. *P=0.002; **P=0.0018; ***P=0.0002; ns, non-significant (repeated measure one-way ANOVA with Tukey's multiple comparisons test, with a single pooled variance).

To determine whether TDP-43 nucleic acid binding and TDP-43 assembly play a role in modulating R-loop formation and maintaining replication function, we tested whether mutations that disrupt these TDP-43 activities affect DNA replication and R-loop accumulation. For these experiments, siRNA-resistant WT or mutant TDP-43 constructs were used to complement TDP-43 KD cells (Fig. 6A,B). Compared with TDP-43 KD alone, expression of the F147/149L mutant, which disrupts RNA/DNA-binding activity (Buratti and Baralle, 2001), failed to rescue replication fork slowing (Fig. 6C, lane 5). A site-directed mutant of the nuclear localization sequence (NLS) that partially prevents TDP-43 nuclear import (Winton et al., 2008) was also unable to rescue replication fork progression (Fig. 6C, lane 7). Similar results were obtained upon complementation with a construct carrying a single site substitution, E17R, which inhibits TDP-43 oligomerization mediated through the N-terminal domain (Wang et al., 2018) (Fig. 6C, lane 6). On the other hand, deletion of the entire C-terminal low complexity domain (ΔC) fully rescued DNA fiber lengths (Fig. 6C, lane 4). In parallel, we observed that expression of F147/149L or E17R mutants did not significantly reduce R-loop levels relative to TDP-43 KD (Fig. 6D, lanes 5 and 6). However, we found a partial reduction in R-loop levels upon expression of the NLS mutant. This reduction was even more significant upon expression of the ΔC mutant (Fig. 6D, lanes 4 and 7), which was similar to the reduction seen after complementation with full length WT TDP-43 (Fig. 6D, lane 3). Collectively, these data suggest that the ability of TDP-43 to prevent R-loop accumulation and maintain replication efficiency requires interactions with RNA (F147/149L mutant) and N-terminally mediated self-assembly (E17R mutant). Moreover, the results with the NLS mutant indicate that the levels of nuclear TDP-43, albeit much reduced upon expression of this mutant (Winton et al., 2008), are sufficient to partially decrease R-loop formation but not sufficient to maintain DNA replication efficiency. Conversely, our findings using the TDP-43 C-terminal domain deletion construct suggest that loss of protein interactions and self-assembly that are mediated through this region do not contribute to the regulation of R-loop formation. This notion was supported by additional experiments showing that two TDP-43 variants that carry mutations in the C-terminal domain associated with ALS, A315T and M337V were able to fully restore replication fork progression (Fig. S5).

### Loss of TDP-43 promotes accumulation of under-replicated regions in mitosis

**Fig. 7.**
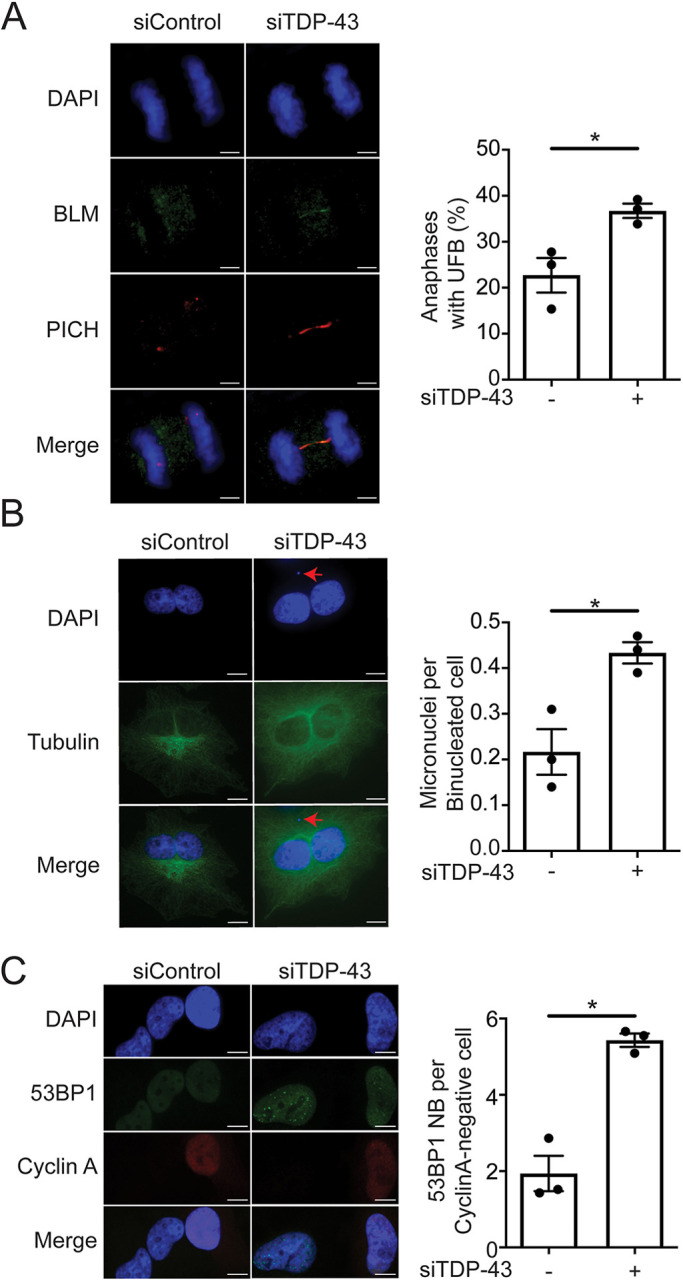
**TDP-43 knockdown induces genomic instability throughout the cell cycle.** (A) HeLa cells were transfected with siControl or siTDP-43 and ultrafine anaphase bridges (UFBs) were visualized using both anti-PICH and anti-BLM antibodies. Representative immunofluorescence images of UFB (left) and percentage of anaphase cells with UFBs (right) from three independent experiments (mean±s.e.m.). Dots represent the means of each independent repeat; *n*≥50 anaphase cells scored for each data set. *P=0.0261 (unpaired *t*-test). (B) siControl and siTDP-43-transfected HeLa cells were treated with cytochalasin B for 24 h to produce binucleated cells and were scored for micronuclei (red arrows) upon DNA staining with DAPI and cytoplasm staining using anti-β-tubulin antibody. Representative immunofluorescence images of micronuclei (left) and frequency of micronuclei per binucleated cell (right) represented as bar plot (mean±s.e.m.) from three independent repeats. Dots represent the means of each independent experiment; *n*≥100 binucleated cells scored for each data set. *P=0.0168 (paired *t*-test). (C) G1-specific 53BP1 nuclear bodies (NB) were scored in HeLa cells transfected with siControl or siTDP43 using anti-53BP1 and anti-cyclin A antibodies. Representative immunofluorescence images of 53BP1 foci formation in HeLa cells (left) and quantification of 53BP1 nuclear bodies per cyclin A-negative G1 phase cell (right) represented as bar plot (mean±s.e.m.) from three independent repeats. Dots represent the means of each independent repeat; *n*≥300 cyclin-A-negative cells scored for each data set. *P=0.0107 (paired *t*-test). Scale bars: 4 µm (A), 10 µm (B,C).

Replication stress can leave chromosomal segments under-replicated, causing an accumulation of under-replicated structures in mitosis, which can in turn impair the proper separation of sister chromatids and lead to chromosomal instability (Liu et al., 2014). Our cell cycle analysis showed that approximately 10-12% of TDP-43 KD cells were able to escape the G2 phase arrest and enter mitosis (Fig. S6A). Moreover, by co-staining these cells with the mitotic marker phospho-H3 (S10) and the DNA damage marker γH2AX, we found that TDP-43 KD cells displaying γH2AX (Fig. S1A, Fig. S6B) were both mitotic and non-mitotic (Fig. S6C). These data are in agreement with our observation that TDP-43-deficient SH-SY5Y cells arrested in prometaphase showed increased chromosomal aberrations, including DNA breaks (Fig. 2D). Interestingly, TDP-43 KD increased the number of mitotic cells containing DNA breaks compared with control cells (Fig. S6C,D). Thus, we sought to investigate whether the replication fork perturbations caused by TDP-43 loss also cause an accumulation of under-replicated regions in anaphase cells, which may in turn lead to the accumulation of DNA breaks that we observed in mitosis (Lukas et al., 2011; Minocherhomji et al., 2015). To this end, we investigated whether TDP-43 loss leads to the accumulation of ultrafine anaphase bridges (UFBs) on the basis of previous findings showing that UFBs are associated with loci that contain under-replicated DNA or aberrant DNA structures that are carried over from S phase into mitosis (Lukas et al., 2011; Minocherhomji et al., 2015) (Fig. 7A). UFBs are not detected by DAPI but can be detected by immunostaining for proteins such as replication protein A (RPA), PLK1-interacting checkpoint helicase (PICH), Bloom syndrome protein (BLM) or proteins of the FANC complex (Baumann et al., 2007; Chan and Hickson, 2009; Chan et al., 2009). Indeed, we found that HeLa cells depleted for TDP-43 displayed a significant increase in the percentage of anaphase cells with UFBs compared with control cells, as assessed by BLM and PICH staining (Fig. 7A). Next, we investigated whether TDP-43 loss leads to the formation of micronuclei and G1-specific 53BP1 nuclear bodies on the basis of previous findings that linked these genomic instability markers to aberrant accumulation of UFBs (Lukas et al., 2011). We found that TDP-43 loss doubled the frequency of binucleated cells with micronuclei (Fig. 7B) as well as dramatically upregulated the number of G1-specific 53BP1 nuclear bodies (Fig. 7C). Collectively, these analyses provide the first evidence that the replication stress phenotype caused by TDP-43 loss in S phase leads to accumulation of under-replicated regions, which transmits through mitosis leading to increased genomic instability. They also suggest that, in addition to the DNA breaks originating upon TDP-43 knockdown in S phase, a fraction of the DNA breaks linked to TDP-43 loss are caused by the breakage of persistent replication intermediates during mitosis.

### Loss of TDP-43 leads to DNA damage and R-loop accumulation in neurons

**Fig. 8.**
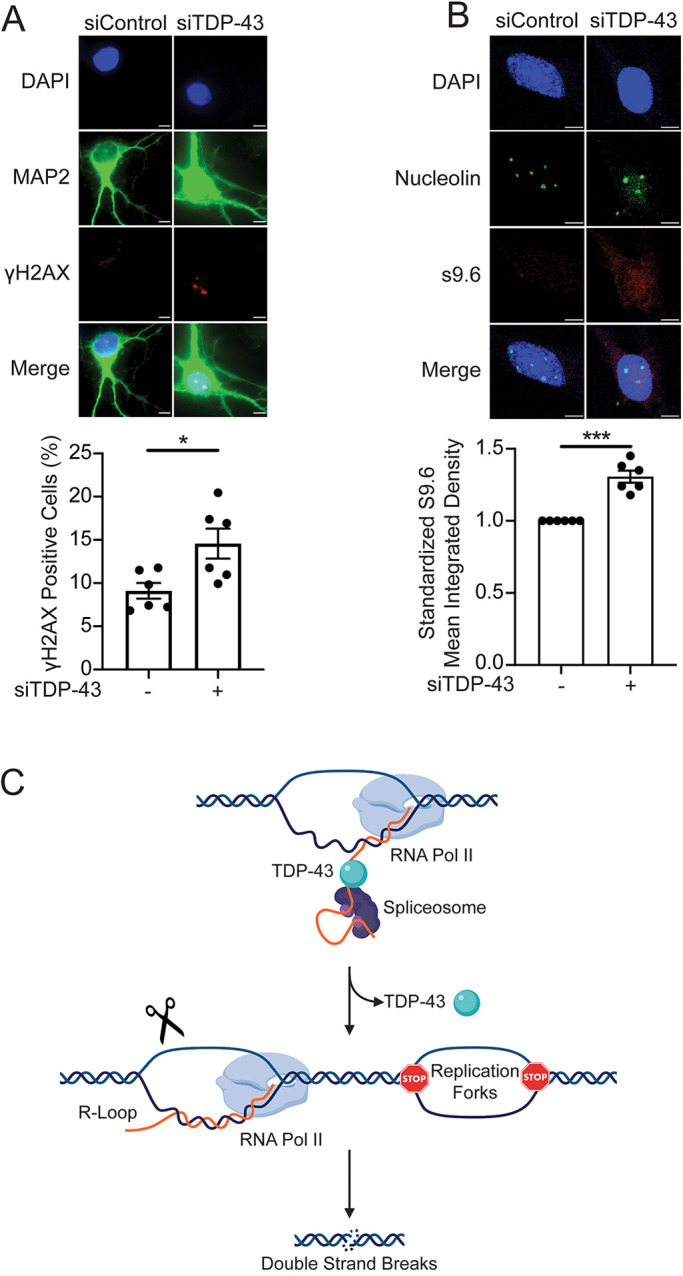
**TDP-43 downregulation increases R-loops and DNA damage in primary neurons and proposed model.** (A) Top: Representative immunofluorescence images of γH2AX staining in murine neuronal cells after transfection with siControl or siTDP-43. Bottom: Percentage of cells with ≥3 γH2AX foci. Data are represented as mean±s.e.m. from two independent experiments performed in triplicate, *n*≥100 cells scored for each data set. *P=0.0271 (paired *t*-test). (B) Top: Representative immunofluorescence staining images with the R-loop-specific antibody S9.6 in murine neuronal cells after transfection with siControl or siTDP-43. Nucleoli were labeled with anti-nucleolin antibody. Nucleolar S9.6 staining was subtracted before S9.6 measurement. Bottom: Quantification of S9.6 intensity signal shown as relative to siControl. *n*≥75 cells scored for each data set. Data are represented as mean±s.e.m. from two independent experiments performed in triplicate. ***P=0.0007; ns, non-significant (paired *t*-test). (C) TDP-43 regulates R-loop formation through a transcription-mediated mechanism. Under normal conditions (top), TDP-43 plays a role in preventing aberrant R-loop accumulation during transcription by associating with nascent RNA. Loss of TDP-43 nuclear function (middle) results in accumulation of R-loops that may be subject to DNA breaks (scissors), both of which are linked to DNA replication stress. Replication stress may include fork stalling or slowing, as indicated by STOP signs, and together with R-loop-associated DNA damage leads to an increase in DNA double-strand breaks (bottom). Scale bars: 5 µm.

We next examined the relevance of our observations to the neurological disorders associated with TDP-43 dysfunction by analyzing the effect of TDP-43 downregulation on R-loop metabolism and DNA damage in neurons. We knocked down TDP-43 in mouse primary neurons using siRNA (Fig. S7) and probed for γH2AX and R-loop accumulation (Fig. 8A,B). TDP-43 downregulation resulted in increased γH2AX staining in neuronal cells (Fig. 8A), as well as an increase in S9.6 staining (Fig. 8B), following the procedures previously used in cycling cells. These data indicate that, as in the other cell lines examined, reduced TDP-43 levels promote the accumulation of R-loops and DNA damage in non-dividing neurons.

## DISCUSSION

TDP-43 joins a growing list of RNA-binding proteins that are important for genome maintenance. However, the exact role of TDP-43 in maintaining genome integrity remains unclear. Here, we provide the first evidence that TDP-43 controls the levels of R-loops and that increased R-loop formation linked to TDP-43 loss drives replication stress and DNA damage. R-loops, consisting of RNA:DNA hybrid structures, play key biological functions, including control of gene expression, transcription termination and immunoglobulin class switch recombination (Skourti-Stathaki et al., 2011; Sun et al., 2013; Yu et al., 2003). However, they can also threaten genome integrity, particularly when replication forks collide with the transcription machinery (Gan et al., 2011; Huertas and Aguilera, 2003; Tuduri et al., 2009; Wahba et al., 2011). Previous studies showed that γH2AX accumulation in cells treated with siRNA targeting TDP-43 can be alleviated by RNase H1 treatment (Hill et al., 2016), an enzyme that specifically digests DNA-associated RNA, suggesting that there might be a link between TDP-43 deficiency, increased DNA damage and accumulation of RNA:DNA hybrid structures. Our data provide direct evidence for this model and show for the first time that TDP-43 deficiency increases R-loop accumulation and leads to replication stress.

Our results provide evidence that TDP-43 regulates R-loop formation and preserves replication fork progression through a transcription-mediated mechanism. Previous studies showed that TDP-43 binds to RNA Pol II, suggesting that TDP-43 is in close proximity to sites of newly transcribed RNA (Das et al., 2007). Based on these previous studies and our own findings, we propose a model whereby TDP-43 associates with nascent RNA during transcription and prevents annealing of RNA to the template DNA strand to form RNA:DNA hybrid structures (Fig. 8C). Loss of TDP-43 would lead to increased formation of R-loops, promoting collisions of RNA pol II transcription and replication machineries during S phase and leading to the replication fork slowing phenotype observed by DNA fiber analysis. In agreement with this model, we also observe a broad distribution of cells in S phase upon TDP-43 KD in our cell cycle and EdU incorporation experiments (Fig. S1B). However, it is possible that R-loop accumulation plays an indirect role in genome-wide DNA replication slowing in TDP-43 knockdown cells through chronic activation of ATR, as recently proposed by Promonet et al. (2020). According to these studies, replication fork pausing at transcription-termination-sites (TTS) prevents head-on collisions by acting as a ‘traffic light’ to help regulate RNA Pol II removal before resuming replication. Therefore, conditions that induce topological stress or abnormal pre-mRNA cleavage could prevent cells from stabilizing forks paused at TTS, resulting in increased DSBs and persistent ATR activation. The genome-wide slowing of replication forks that we observe in TDP-43 deficient cells could be caused by persistent ATR activation by R-loops at TTS. In addition, our findings do not rule out the alternative possibility that the effects caused by TDP-43 loss are compounded by aberrant expression of key targets controlled by TDP-43 that regulate R-loop metabolism.

TDP-43 is a modular protein composed of independently folded domains and a mostly disordered C-terminal region. Work from our groups and others are providing improved understanding of how each domain and the interactions between the different regions orchestrate different TDP-43 activities. For instance, the C-terminal tail promotes interactions with other heterogeneous nuclear ribonucleoproteins, which are necessary for the regulation of splicing in several systems (D'Ambrogio et al., 2009) and required for TDP-43 autoregulation (Ayala et al., 2011). In addition, this domain promotes TDP-43 self-assembly through phase separation (Conicella et al., 2016; Schmidt and Rohatgi, 2016). However, loss of the C-terminal region does not affect RNA- and DNA-binding activity (Ayala et al., 2005) or TDP-43 oligomerization mediated by the N terminus or RRMs (French et al., 2019). Our findings that the C-terminal domain is not required for modulating R-loop or DNA replication by TDP-43 suggests that the associated regulatory mechanism is different from that controlling splicing and does not depend on C-terminal domain-mediated phase separation.

By contrast to the C-terminal tail, we found that the N-terminal domain of TDP-43 is a key regulator R-loop metabolism. This region mediates TDP-43 oligomerization and is regulated by phosphorylation of Ser48 located in the N-terminal domain (Afroz et al., 2017; Mompeán et al., 2017; Wang et al., 2018; Zhang et al., 2013). Future studies should focus on defining whether the N terminus participates in controlling R-loops through interaction with novel partners or by promoting self-assembly and whether these functions are regulated via Ser48 phosphorylation.

Our studies open the tantalizing scenario that TDP-43 function in R-loop regulation is compromised in TDP-43 proteinopathies. Supporting this model, we observed R-loop accumulation and increased DNA damage in neurons after TDP-43 downregulation. The increased genomic instability linked to R-loop formation in neuronal cells treated with siRNA targeting TDP-43 probably relies on other mechanisms that increase DNA breaks at R-loop structures in a replication-independent fashion in post-mitotic cells. For instance, sites of transcription blocks caused by R-loops may be subject to excision by the transcription-coupled nucleotide excision repair nucleases XPG and XPF leaving single-strand DNA lesions. The single-strand DNA lesions or gaps can either cause replication fork collapse and DSB formation in S phase, or can be converted into DSBs in a replication-independent fashion by cleavage of both strands of the R-loop by XPG and XPF (Sollier et al., 2014; Shivji et al., 2018; Yasuhara et al., 2018). Interestingly, we also found that TDP-43 overexpression, which is associated with cytoplasmic aggregation as observed in patient cells (Arai et al., 2006; Neumann et al., 2006), increases R-loops and results in significant DNA damage. These results suggest that TDP-43 aggregation, by sequestering soluble nuclear TDP-43, leads to a loss-of-function phenotype, increasing the levels of R-loop formation and genomic instability. Our findings are in agreement with previous observations that TDP-43 nuclear clearance correlates with increased DNA breaks in the spinal cord of ALS patients (Mitra et al., 2019). The importance of DNA damage and R-loop accumulation in ALS and FTD pathogenesis is further highlighted by additional ALS- and FTD-associated mutations. Senataxin is an RNA:DNA helicase that resolves R-loops whose function is important to preserve genomic stability (Mischo et al., 2011; Skourti-Stathaki et al., 2011). Mutations in *SETX* are linked to juvenile ALS (ALS type 4) (Avemaria et al., 2011; Chen et al., 2004), suggesting that neurodegeneration may be caused by defects in R-loop metabolism. In addition, the hexanucleotide expansion of G_4_C_2_ in *C9Orf72* is the most common genetic mutation in ALS and FTD (DeJesus-Hernandez et al., 2011; Renton et al., 2011). Models of the *C9Orf72* mutation show increased R-loop accumulation, and spinal cord neurons from ALS *C9Orf72* human tissue show elevated levels of R-loops and increased DNA breaks (Walker et al., 2017). These findings, along with our results, strongly suggest that increased R-loop formation and genomic instability are previously unappreciated drivers of neurodegeneration in ALS and FTD, and open new avenues to understand how preventing R-loop formation and DNA damage might be exploited therapeutically to treat these devastating neurological disorders.

## MATERIALS AND METHODS

### Reagents

All reagents were purchased from Sigma-Aldrich unless otherwise noted.

### Cell culture and transfection with siRNA and expression vectors

Cells were cultured at 37°C in 5% carbon dioxide and atmospheric oxygen. HeLa cells were obtained from ATCC and cultured in high-glucose Dulbecco's modified Eagle's medium (DMEM) containing 10% heat-inactivated fetal bovine serum (FBS) and 100 μg/ml penicillin/streptomycin. SH-SY5Y cells were obtained from ATCC and cultured in 1:1 DMEM:Nutrient Mixture F-12 containing 10% heat-inactivated FBS and 100 μg/ml penicillin/streptomycin.

Transient TDP-43 gene depletion in HeLa cells was performed using siRNA targeting TDP-43 (siTDP-43, GCAAAGCAAGAUGAGCCUdTdT; Dharmacon) at a final concentration of 100 nM and oligofectamine transfection reagent (Thermo Fisher Scientific) according to the manufacturer's protocol. Briefly, in one tube, 4 μl of oligofectamine were combined with 15 µl of Opti-MEM (Thermo Fisher Scientific) and incubated for 6 min. Concurrently, 5 µl of 20 µM siTDP-43 were mixed with 180 μl Opti-MEM. After the 6 min incubation, the two reactions were mixed and incubated for 20 min before adding to 10^5^ cells seeded in a six-well plate 24 h previously. After 4 h incubation, the reaction was stopped by adding 1 ml of Opti-MEM with 30% FBS. Two transfections with siTDP-43 were carried out with a 24 h interval between them and experiments were performed 24 h after the second transfection. A non-targeting control pool of siRNA (Dharmacon D-001810-01-05) was used as a control (siControl) in parallel with siTDP-43 transfections.

Transient TDP-43 gene depletion in SH-SY5Y cells was performed using a second siTDP-43 (GGUGGUGCAUAAUGGAUAUdTdT, Dharmacon) (Yu et al., 2012) at a final concentration of 100 nM and lipofectamine RNAiMAX transfection reagent (Life Technologies) according to the manufacturer's protocol. Briefly, in one tube, 9 μl of RNAiMAX was combined with 150 μl of Opti-MEM (Thermo Fisher Scientific). Concurrently, 5 μl of 20 µM siTDP-43 were mixed with 150 μl Opti-MEM. The two reactions were mixed and incubated for 5 min before adding 250 μl of the reaction to 2×10^5^ cells seeded in a six-well plate 24 h previously. The reaction was incubated with cells for 24 h before changing the medium. Three successive transfections 24 h apart were carried out for TDP-43 transient knockdown in SH-SY5Y cells; experiments were performed 24 h after the third transfection. A non-targeting control pool of siRNA (Dharmacon D-001810-01-05) was used as a control (siControl) in parallel with siTDP-43 transfections.

Transfection of siRNA-resistant FLAG-WT and mutant FLAG-TDP-43 constructs (D'Ambrogio et al., 2009) as well as transfections of GFP-WT and GFP-tagged nuclease dead D145N mutant RNase H1 constructs (Parajuli et al., 2017) were performed using Mirus TransIT-LT1 Transfection Reagent (MirusBio) according to the manufacturer's protocol. Briefly, 250 μl of Opti-MEM (Thermo Fisher Scientific), 1 μg plasmid DNA (unless otherwise noted) and 7.5 μl of TransIT-LT1 reagent were gently mixed together and incubated for 30 min before adding to cells at ≥80% confluency, 24 h after the last transfection with siRNA. Transfected cells were incubated with TransIT-LT1 reactions for 24 h before harvesting cells.

### Primary neuron culture

Hippocampi from embryonic day 18 CD1 mice were dissected in Hanks Balanced Salt Solution, triturated using a fire-polished Pasteur pipette, digested with 0.25% trypsin and 0.02 mg/ml DNase, and resuspended in plating medium containing 10% heat-inactivated horse serum. Viable cells were counted by trypan blue exclusion and cell density adjusted to 25,000 cells/cm^2^. Cells were plated onto glass coverslips coated with poly-L-lysine and incubated at 37°C and 5% carbon dioxide. Cells were allowed to attach for 2 h, after which plating medium was removed and replaced with maintenance medium containing Neurobasal Medium and B27 supplement. After 4-5 days *in vitro* (DIV), medium was replaced with Accell siRNA delivery medium supplemented with B27 and containing 1 µM siRNA directed against TDP-43 (Accell siRNA Smartpool mouse *Tardbp*, Dharmacon). Control conditions were treated with Accell Non-targeting Pool siRNA. Neurons were cultured for five additional days and then fixed with either 100% methanol for 10 min at 4°C or 4% paraformaldehyde for 10 min at room temperature (RT) and rinsed with phosphate-buffered saline (PBS). All animal experiments were performed according to approved guidelines.

TDP-43 downregulation in primary neurons was quantified by real-time PCR. RNA and cDNA preparation were carried out using Trizol and PureLink RNA mini kit (ambion) and Superscript III First-Strand Synthesis System (Invitrogen), respectively. PCR was performed using iTaq Universal SYBR Green Supermix (Bio-Rad) with primers for mouse *Tardbp*: mTDPex2FW, 5′-GGGGCAATCTGGTATATGTTG-3′; mTDPex3RV, 5′-TGGACTGCTCTTTTCACTTTCA-3′; mTDPex4FW, 5′-TGGTGTGACTGTAAACTTCCC-3′; mTDPex5RV, 5′-GACATCTACCACTTCTCCATACTG-3′*.* Primers 36B4 FW, 5′-CACTGGTCTAGGACCCGAGAAG-3′, and 36B4 RV, 5′-GGTGCCTCTGAAGATTTTCG-3′, were used to quantify mouse *36B4* as reference. Real-time PCR was carried out on a CFX96 Bio-Rad instrument.

### Isolation of nuclear fraction

Cellular fractionation was carried out to isolate nuclei from HeLa cells (approximately 20×10^6^) collected 24 h post siRNA treatment (Ayala et al., 2006). Cell pellets were resuspended in 3 ml of buffer A (10 mM HEPES pH 7.9, 1.5 mM MgCl_2_, 10 mM KCl, 0.5 mM TCEP, EDTA-free protease inhibitor cocktail). The cell membrane was disrupted with 20 strokes of a pre-chilled Dounce homogenizer using a tight pestle. Nuclei were pelleted by centrifugation (1000 ***g*** for 5 min) and rinsed in buffer A. All steps were performed at 4°C. Total DNA was isolated from the nuclear pellet using QIAamp DNA mini kit (Qiagen) according to manufacturer's instructions. For slot blot analysis of S9.6 and double-strand (ds)DNA, DNA (0.25 and to 0.5 μg) was loaded on Hybond N+ (GE-Healthcare) using a Bio-Dot SF microfiltration apparatus (Bio-Rad) according to the manufacturer's instructions before crosslinking (120 mJ/cm^2^). Membranes were blocked in 5% blotting grade blocker (Bio-Rad) in TBST (Tris-buffered saline containing 0.1% Tween) and probed with S9.6 (1:1000; Kerafast ENH001) or dsDNA (1:2000; Abcam ab27156) in 2% blotting grade blocker in TBST overnight at 4°C. Blots were quantified by near-infrared western blot detection (LI-COR Biosciences). RNaseH treatment was carried out with 0.5 μg total DNA and 5 U RNase H1 (New England Biolabs) overnight at 37°C.

### Immunoblotting and antibodies

Protein detection by western blot analysis was performed as previously described (Quinet et al., 2019). Briefly, cells were washed with PBS before protein extraction in lysis buffer (50 mM Tris-HCl pH 7.5, 20 mM NaCl, 1 mM MgCl_2_, 0.1% SDS), 250 U/ml Benzonase (71206, Novagen), and 1× PhosStop (Sigma-Aldrich 4906845001) for 20 min on ice. Following centrifugation, supernatant lysate was quantified using Pierce BCA protein assay (Thermo Scientific 23227) according to the manufacturer's instructions. Lysates were denatured in 1× NuPAGE LDS buffer (NP0007, Thermo Fisher Scientific) and 200 mM DTT at 100°C for 5 min. Denatured lysates were evenly loaded into SDS-PAGE NuPAGE Novex 4-12% Bis-Tris gels (NP0322BOX, Thermo Fisher Scientific) with NuPAGE MES SDS running buffer (NP0002, Thermo Fisher Scientific). Electrophoresed proteins were transferred to 0.45 μm pore nitrocellulose membranes (10600002, GE Healthcare Life Sciences) via wet transfer in 1× Tris/glycine buffer (161-0732, Bio-Rad) with 20% methanol for immunoblotting.

Membranes developed with films were blocked with 5% milk (170-6404, Bio-Rad) and 0.1% Tween-20 (P1379, Sigma) in PBS for 1 h at RT. HRP-conjugated secondary antibodies (1:10,000; goat anti-rabbit IgG PI31460, Fisher) (1:10,000; goat anti-mouse IgG 62-6520, Life Tech) were used and detected using ECL (32106, Pierce) according to the manufacturer's instructions. Membranes developed using the Li-COR system were blocked with 5% BSA (170-6404, Bio-Rad) in PBS for 1 h at RT. Secondary antibodies used were IRDye Infrared (1:10,000; goat anti-rabbit 926-68071, Li-COR) (1:10,000; goat anti-mouse 926-32210, Li-COR) and were detected with Odyssey CLx (Li-COR). Empiria Studio software (version 1.0.1.53, LI-COR) was used for semi-quantitative determination of protein expression in western blot analyses. The following primary antibodies were used: TDP-43 (1:2000; 107822AP, Fisher Scientific), GAPDH (1:20,000; ab181602, Abcam), phospho-H3 (S10) (1:1000; 06-570, Millipore), γH2AX (S139) (1:1000; 05-636, Millipore), RNase H1 (1:5000; H00246243-M01, Novus Bio).

### DNA fiber assay

DNA fiber assay was performed as previously described (Quinet et al., 2019). Briefly, asynchronous exponential growing cells were sequentially labeled with two thymidine analogs: 20 μM 5-iodo-2′-deoxyuridine (IdU, Millipore Sigma) for 30 min followed by 200 μM 5-chloro-2′-deoxyuridine (CldU, Millipore Sigma) for 30 min. Cells were washed twice with PBS after each pulse labeling with analog. After the pulse labels, cells were collected and resuspended in PBS at a concentration of 1500 cells/μl. A total of 2 μl of this cell solution was mixed with 6 μl of lysis buffer (200 mM Tris-HCl pH 7.5, 50 mM EDTA, 0.5% SDS) on a positively charged glass slide. After incubating for 4 min, the slides were tilted at a 20–45° angle, allowing the cells to spread evenly at a constant speed. The resulting DNA spreads were air dried for 10 min, fixed in 3:1 methanol:acetic acid for 5 min and stored at 4°C.

For immunostaining of the thymidine analogs, the DNA fibers were denatured with 2.5 M HCl for 1 h at RT, washed with PBS and blocked with 5% BSA in PBS for 1 h at 37°C. DNA immunostaining was performed with rat anti-BrdU antibody (1:100; Abcam, Ab6326) for CldU and mouse anti-BrdU antibody (1:50; Becton Dickson, 347580) for IdU in a humid chamber at 37°C for 90 min. Slides were then washed three times with 0.1% Tween-20 in PBS for 5 min each at RT. The following secondary antibodies were used: anti-mouse AlexaFluor 488 (1:100; Thermo Fisher Scientific, A21470) and anti-rat AlexaFluor 546 (1:100; Thermo Fisher Scientific, A21123) at RT for 1 h. Slides were again washed three times with 0.1% Tween-20 in PBS for 5 min each at RT. The slides were air dried and mounted with Prolong Gold Antifade reagent (Invitrogen, P36930).

Images were sequentially acquired for the red and green channels at RT on a fluorescent microscope using the 63× objective (Leica DMU 4000B; 63×/1.40-0.60 NA oil). At least 10 images per condition were taken across several sections of the slide, using a single channel to select for regions of interest to prevent potential bias. A minimum of 150 tracts were scored per data set, where each tract must include an unambiguously defined beginning and end for both colors. The DNA tract lengths were measured using ImageJ and the pixel length values were converted into micrometers using the scale bars created by the microscope. All DNA fiber experiments were performed three times independently.

### Neutral comet assay

Neutral comet assays were performed using CometAssay (Trevigen) according to the manufacturer's protocol with minor modifications (Carvajal-Maldonado et al., 2019). Upon harvesting, cells were suspended at 3×10^5^ cells/ml in cold PBS before combining with LMAgarose (Trevigen, 4250-50-050-02), spread onto a comet slide (Trevigen, 4250-200-03) and allowed to dry. Slides were placed in lysis solution (Trevigen, 4250-050-01) at 4°C for 1 h. Lysed slides were immersed in 1× TBE buffer (0.1 M Tris base, 0.1 M boric acid, 2.5 mM EDTA) for 30 min before electrophoresis at 25 V for 30 min at 4°C. Slides were washed in DNA precipitate solution (1 M ammonium acetate, 95% EtOH) for 30 min, followed by a fixing step in 70% ethanol for 30 min, and dried overnight at RT. Comets were stained with 1× SYBR Gold (Thermo Fisher Scientific) for 30 min. Images were acquired with a fluorescence microscope (Leica DMU 4000B; 63×/1.40-0.60 NA oil) with a Leica DFC345FX camera. At least 150 comets were scored in each experiment using the OpenComet plugin in the ImageJ analysis software.

### Cell cycle analysis

Cell cycle analysis was performed using Click-iT EdU Imaging Kit (C10337, Invitrogen) according to the manufacturer's protocol. Briefly, asynchronous cells were treated with 10 μM 5-ethynyl-2′-deoxyuridine (EdU; E10187, Thermo Fisher Scientific) for 30 min prior to harvesting to label nascent DNA. Cells were washed and trypsinized before fixing in 3.7% formaldehyde for 10 min. Fixed cells were blocked in 1% BSA/PBS at RT for 10 min before permeabilization by 0.5% saponin in the dark for 30 min at RT. Permeabilized cells were then incubated with the Click-iT cocktail in the dark for 30 min at RT before DNA staining with DAPI solution (1% BSA in PBS, 0.1 mg/ml RNase A, 2 μg/ml DAPI) for 30 min in the dark. Samples were run through flow cytometry via FACSCanto II (BD Biosciences) and data were analyzed and visualized using FlowJo software.

### Immunofluorescence microscopy

Detection of S9.6 was performed as described previously (Hamperl et al., 2017) with minor modifications. Briefly, transfected cells seeded on glass coverslips were fixed with 100% ice-cold methanol for 10 min at −20°C. Fixed cells were treated with 6 μg/ml RNase A (12091-039, Invitrogen) in RNase A buffer (10 mM Tris-HCl, 5 mM NaCl, 5 mM EDTA) for 30 min at 37°C to avoid nonspecific binding of single-stranded RNA outside R-loops. For treatments with the RNase H endonuclease, cells were also treated with 7 μg/ml RNase H nuclease (M0297, NEB) for 4 h at 37°C. Cells were then washed in PBS and blocked in 5% BSA diluted in PBS for 30 min at 37°C, followed by incubation with the S9.6 primary antibody (1:500; ENH001, Kerafast) and rabbit anti-nucleolin primary antibody (1:2000; ab22758, Abcam) for 2 h in a humid chamber at 37°C. Secondary antibodies anti-mouse Alexa Fluor 546 (1:1000; Thermo Fisher Scientific, A21123) and anti-rabbit Alexa Fluor 488 (1:1000; Thermo Fisher Scientific, A11034) and DAPI were incubated with cells for 1 h in a humidity chamber at 37°C. Finally, coverslips were mounted onto glass slides using ProLong Gold (P36930, Invitrogen). Images were taken using a 63× objective with a confocal fluorescent microscope (Leica DMU 4000B; 63×/1.40-0.60 NA oil). Only nuclear staining was used to quantify R-loop accumulation, and all cytoplasmic and nucleoli staining was removed before analysis. We subtracted cytoplasmic and nucleolar staining, as determined by co-staining with nucleolin. Images were analyzed using an ImageJ macro that sequentially subtracted nucleolin staining, detected and outlined nuclei based on DAPI staining and subsequently measured S9.6 staining in nuclei only. In order to minimize staining-based sample deviation, the mean value for each individual experiment condition was standardized to the negative control.

TDP-43 immunofluorescence analysis was performed following the protocol described above and using a specific primary antibody targeting TDP-43 (1:2000; 107822AP, Fisher Scientific).

### Chromosome spreads

Chromosomes spreads were prepared as described previously (Lemaçon et al., 2017). Briefly, 48 h after siControl or siTDP-43 treatment, SH-SY5Y cells were treated with 10 μM nocodazole for 4 h. Arrested cells were trypsinized and resuspended in warm hypotonic solution (75 mM KCl, 5% FBS) for 10 min at 37°C. Then, 500 μl of 4°C fixing solution (3:1 ethanol:acetic acid) was added to cells while gently vortexing. Fixed cells were washed three times with 4°C fixation buffer and stored at 4°C for at least 24 h before spreading. Chromosomes were spread by dropping onto chilled glass slides. Slides were air dried and mounted with Prolong Gold Antifade (Invitrogen) and DAPI. Images were acquired with a fluorescence microscope (Leica DMU 4000B; 63×/1.40-0.60 NA oil). Captured images were analyzed with ImageJ. At least 50 metaphase spreads were assessed per sample. Chromosomes were assessed for both chromatid breakage and end-to-end fusions. For each condition, the total number of aberrations counted was divided by the total number of metaphase spreads assessed to determine the average number of aberrations per cell.

### Ultrafine anaphase bridge detection

UFBs were detected as previously described (Bizard et al., 2018). Briefly, 48 h after siControl or siTDP-43 treatment, HeLa cells were treated with 40 ng/ml nocodazole for 4 h to arrest mitotic cells in prometaphase. Cells were released from nocodazole for exactly 45 min and incubated with fresh medium. Arrested cells were simultaneously extracted and fixed with co-extraction buffer (20 mM Pipes, 10 mM EGTA, 0.2% Triton X-100, 1 mM MgCl_2_, 4% PFA) for 15 min at RT, washed three times in PBS, and then permeabilized and blocked in BSA-T (5% BSA, 0.5% Triton) overnight at 4°C. Immunostaining of the UFB was performed with rabbit anti-BLM antibody (1:200; Abcam, Ab2179) and mouse anti-PICH antibody (1:100; abnova, H00054821-B01P) in a humid chamber at 4°C overnight. Slides were then washed three times in PBS for 5 min each at RT. Secondary antibodies anti-mouse Alexa Fluor 546 (1:1000; Thermo Fisher Scientific, A21123) and anti-rabbit Alexa Fluor 488 (1:1000; Thermo Fisher Scientific, A11034) were incubated with cells for 1 h at RT in the dark. Slides were again washed three times in PBS for 5 min each at RT. Nuclei were stained with 0.05 μg/ml DAPI. The slides were mounted with Prolong Gold Antifade reagent (Invitrogen, P36930). Images were captures at 63× objective and UFBs were only counted if the following conditions were met: the cytoplasm was ovoid, with the DNA from each future daughter cell within the same cytoplasm without overlapping chromosomes; there were clear bridges present; and the UFB stained for both overlapping PICH and BLM. At least 50 anaphases were counted per condition.

### G1-specific 53BP1 nuclear bodies

At 48 h after siControl or siTDP-43 treatment, HeLa cells were fixed with 4% PFA for 10 min, washed three times in PBS, permeabilized in 0.5% Triton for 10 min at RT, before blocking in BSA-T for 1 h at RT. Immunostaining of the 53BP1 bodies was performed with rabbit anti-53BP1 antibody (1:1000; Novus Biologicals, NM100-304S) and mouse anti-cyclin A antibody (1:100; Santa Cruz, SC-271682) in a humid chamber at 4°C overnight. Slides were then washed three times in PBS for 5 min each at RT. Secondary antibodies anti-mouse Alexa Fluor 546 (1:1000; Thermo Fisher Scientific, A21123) and anti-rabbit Alexa Fluor 488 (1:1000; Thermo Fisher Scientific, A11034) were incubated with cells for 1 h at RT in the dark. Slides were again washed three times in PBS for 5 min each at RT. Nuclei were stained with 0.05 μg/ml DAPI. The slides were mounted with Prolong Gold Antifade reagent (Invitrogen, P36930). Images were captures at 40× objective, and at least 300 cyclin A-negative cells were analyzed per condition.

### Micronuclei assay

At 48 h after siControl or siTDP-43 treatment, HeLa cells were treated with 4 μg/ml cytochalasin B (Millipore Sigma, C6762) for 24 h. After treatment, cells were fixed with 4% PFA for 10 min, washed three times in PBS, permeabilized in 0.5% Triton for 10 min at RT, before blocking in BSA-T for 1 h at RT. Immunostaining of cytoplasm was performed with rabbit anti-β-tubulin antibody (1:200; Abcam, ab6046) in a humid chamber at 4°C overnight. Slides were then washed three times in PBS for 5 min each at RT. Secondary antibody anti-rabbit Alexa Fluor 488 (1:1000; Thermo Fisher Scientific, A21123) were incubated with cells for 1 h at RT in the dark. Slides were again washed three times in PBS for 5 min each at RT. Nuclei were stained with 0.05 μg/ml DAPI. The slides were mounted with Prolong Gold Antifade reagent (Invitrogen, P36930). Images were captures at 40× objective, and at least 100 binucleated cells were analyzed per condition.

### RNA:DNA immunoprecipitation quantitative PCR assay

DRIP-qPCR was performed as described (Promonet et al., 2020). Briefly, 5×10 cells were lysed in 0.5% SDS in Tris-EDTA buffer pH 8.0 containing Proteinase K overnight at 37°C. Total DNA was isolated by phenol/chloroform/isoamyl alcohol extraction followed by standard ethanol precipitation. One-third of total DNA was fragmented by restriction enzyme cocktail (*Msel*, *Alul*, *Mbol* and *Ddel*) overnight at 37°C. Digested DNA was purified by phenol/chloroform/isoamyl alcohol extraction and ethanol precipitation, and quantified by Nanodrop. Digested DNA (8 μg) was treated overnight with RNase H1, followed by phenol/chloroform/isoamyl alcohol extraction and ethanol precipitation as a negative control. Digested DNA (4 μg) was diluted in binding buffer (10 mM NaPO_4_ pH 7.0, 0.14 M NaCl, 0.05% Triton X-100) and incubated with 10 μg of S9.6 antibody overnight at 4°C on a rotator. DNA/antibody complexes were combined with Protein A/G agarose beads prewashed with binding buffer and incubated for 2 h at 4°C. Immunoprecipitated RNA:DNA was eluted by incubation with elution buffer (50 mM Tris pH 8.0, 10 mM EDTA, 0.5% SDS) containing Proteinase K at 55°C for 45 min on a rotator. The eluent was precipitated by phenol/chloroform/isoamyl alcohol extraction and ethanol precipitation. Enrichment of RNA:DNA hybrids was quantified by qPCR using the following specific primers: SLC35B2 forward, 5′-AAGTCTTGCCCTAGCTGTGCT-3′; SLC35B2 reverse, 5′-GCCTACACCGCTTGTGCTTTT-3′; RPL13A forward, 5′-AATGTGGCATTTCCTTCTCG-3′; RPL13A reverse, 5′-CCAATTCGGCCAAGACTCTA-3′; SNRPN forward, 5′-GCCAAATGAGTGAGGATGGT-3′; SNRPN reverse, 5′-TCCTCTCTGCCTGACTCCAT-3′

### Statistical analysis

Statistical analyses were performed using Prism (GraphPad software). Statistical significances are indicated in figure legends.

## Supplementary Material

Supplementary information
