# Supplementary material for: TDP-43 dysfunction results in R-loop accumulation and DNA replication defects
Source: J Cell Sci. 2020 Oct 30;133(20):jcs244129. doi: 10.1242/jcs.244129 (PMC7648616; doi:10.1242/jcs.244129)
Supplement: Supplementary information [file joces-133-244129-s1.pdf]

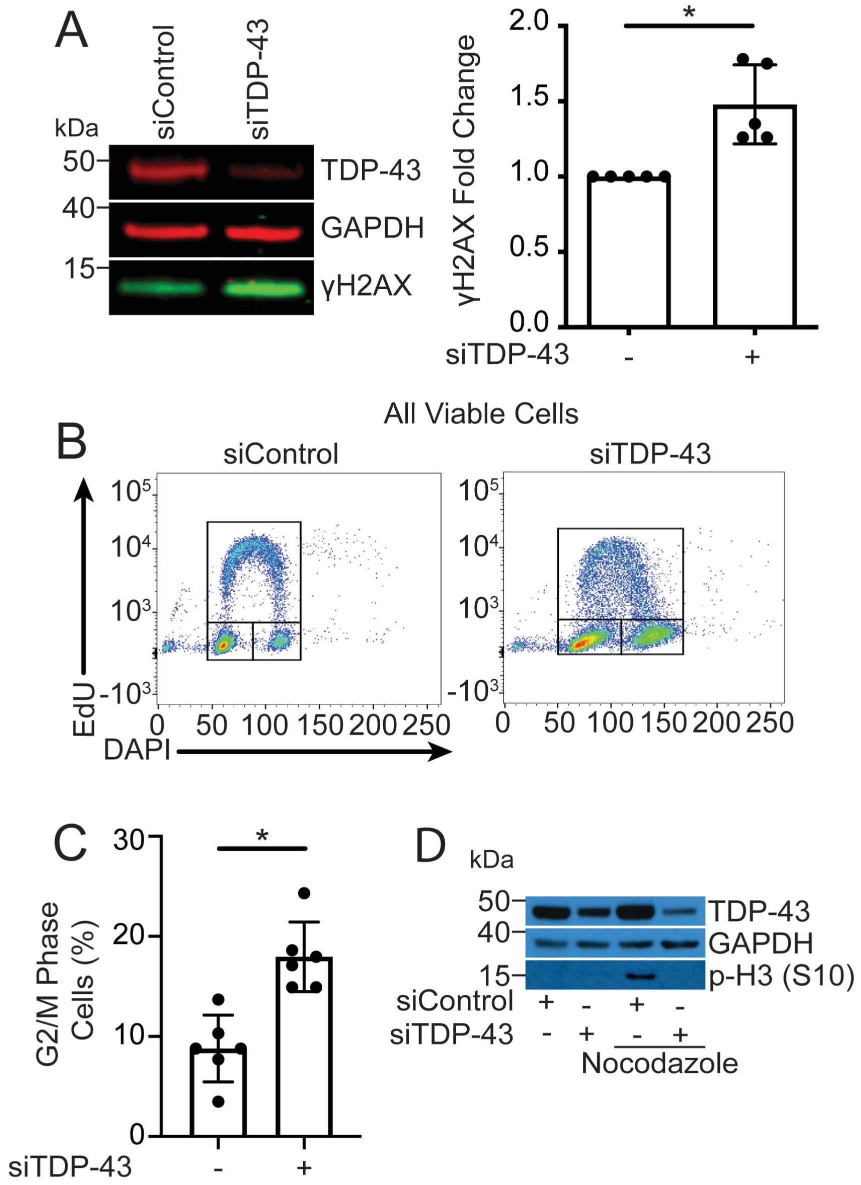

Figure S1: TDP-43 Knock Down Increases DNA Damage. (A) Left, protein expression of TDP-43 and the DNA damage marker γH2AX after TDP-43 siRNA knockdown (siTDP-43) in HeLa cells. Right, quantification of γH2AX expression levels after TDP-43 knockdown in HeLa cells (mean ± SEM) from three independent experiments. Statistics: paired t-test; *P = 0.0151. (B) Cell cycle profiles detected by flow cytometry in siControl and siTDP-43 transfected HeLa cells. The thymidine analog EdU was added to cell culture media for 30 min prior to cell harvesting to monitor cells distribution in all phases of the cell cycle. (C) Quantification of cells in G2/M phase after TDP-43 knockdown in HeLa cells (mean ± SEM) from 6 independent experiments. Statistics: paired t-test; *P = 0.0128. (D) Protein expression of TDP- 43 and the mitosis marker phospho-H3 (S10) after TDP-43 siRNA knockdown and 4 h 10 μM nocodazole treatment in HeLa cells by western blot. A representative western blot from three experiments is shown.

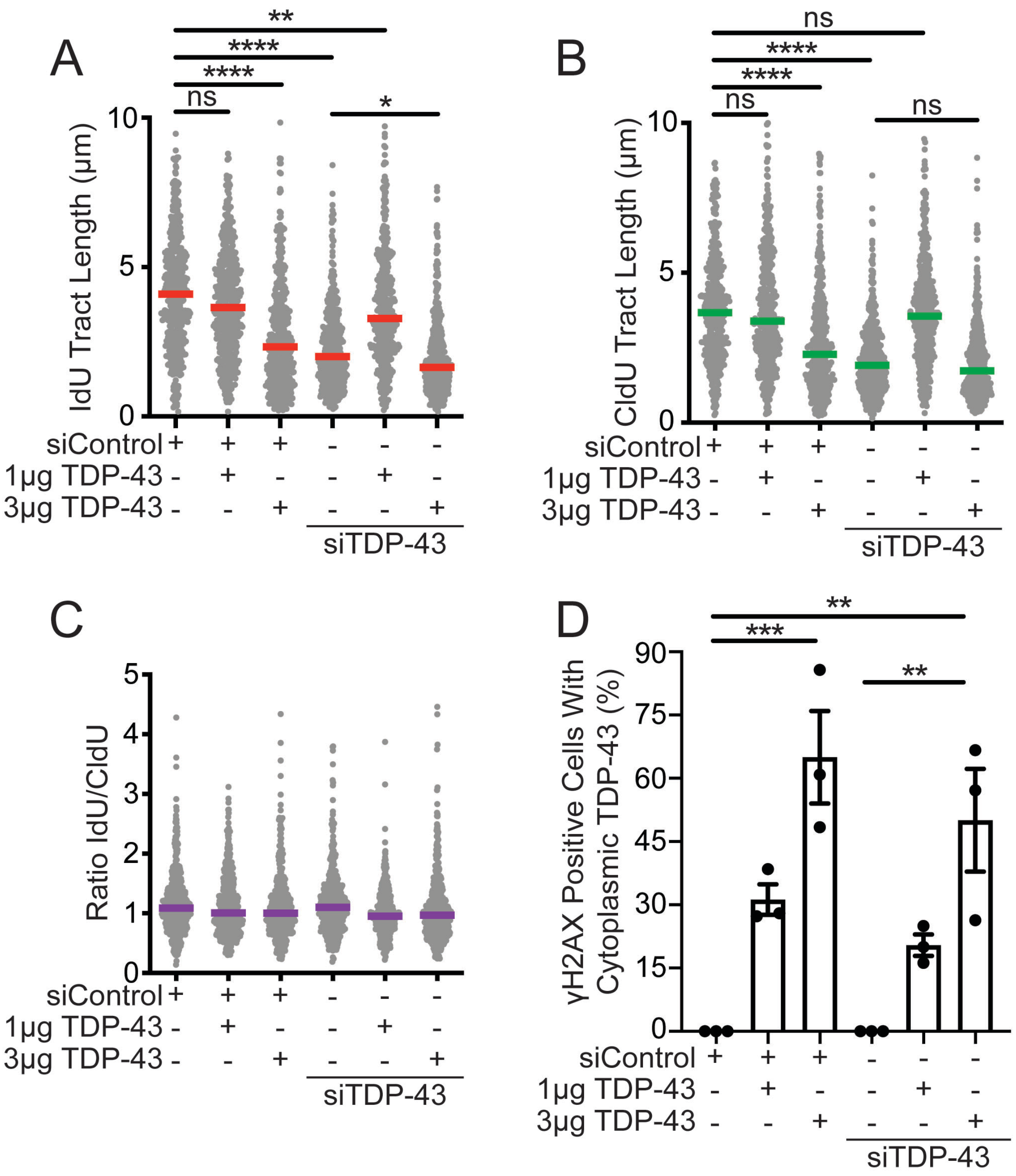

Figure S2: TDP-43 Knock Down and Overexpression Induces Replication Stress. Size distribution of IdU tracts (A), CldU tracts (B), and IdU/CldU tract length ratio (C) in HeLa cells after transfection with siControl or siTDP-43 and siRNA-resistant WT FLAG-TDP-43 vector from experiments shown in Figure 1D. Data are pooled from three independent experiments and shown as dot plot. Bars represent the median, and median values ± SEM are indicated above each condition. *n* ≥ 150 tracts scored for each data set. Statistics: Kruskal-Wallis test with Dunn's multiple comparisons test; ns, non-significant, **P*=0.0424, ***P*=0.0011, *****P* < 0.0001. (D) Quantification of cells with ≥ 3 γH2AX foci represented as bar plot after TDP-43 depletion and overexpression. Data are represented as mean ± SEM from three independent experiments, *n* ≥ 100 cells scored for each data set. ***P*=0.0016, ****P*=0.0002 (paired t-test).

Journal of Cell Science • Supplementary information

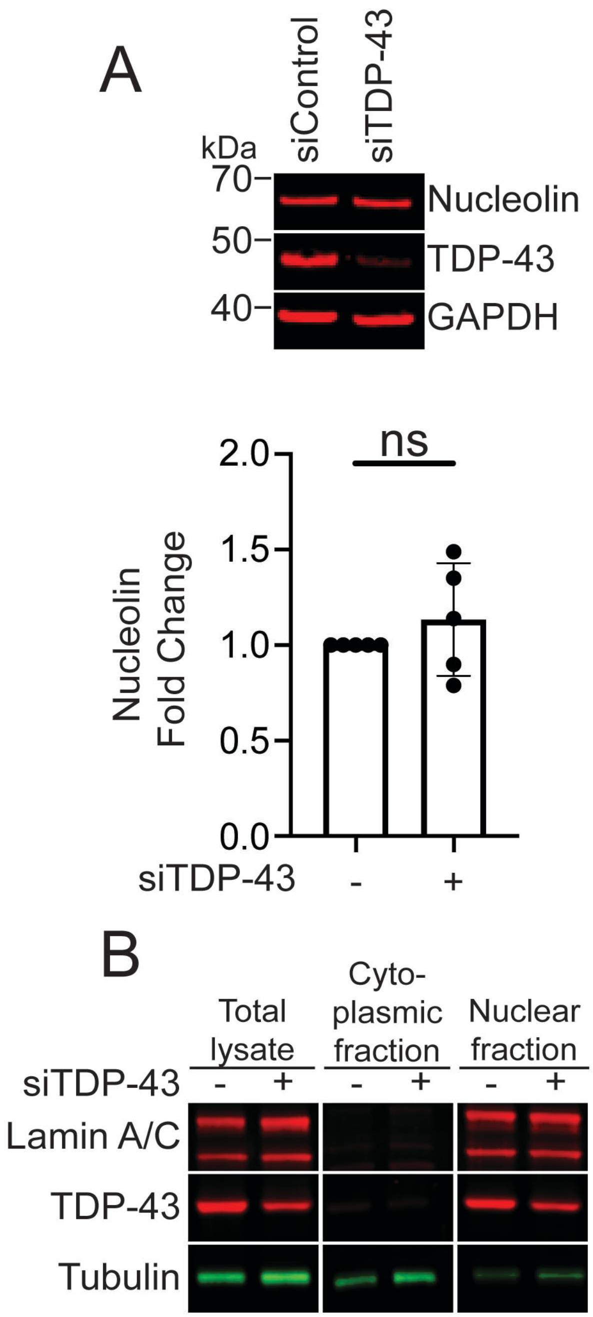

Figure S3: TDP-43 Knock Down Does Not Alter Nucleolin Expression. (A) Top, protein expression of TDP-43, GAPDH, and nucleolin after TDP-43 siRNA knockdown (siTDP-43) in HeLa cells. Bottom, quantification of nucleolin expression levels after TDP-43 knockdown in HeLa cells (mean ± SEM) from five independent experiments. Statistics: paired t-test; ns = not significant. (B) Representative immunoblot of HeLa fractionation after TDP-43 (siTDP-43) or control siRNA treatment. Total lysate, cytoplasmic and nuclear fractions were probed with antibodies recognizing lamin A/C, TDP-43 or β-tubulin.

Journal of Cell Science • Supplementary information

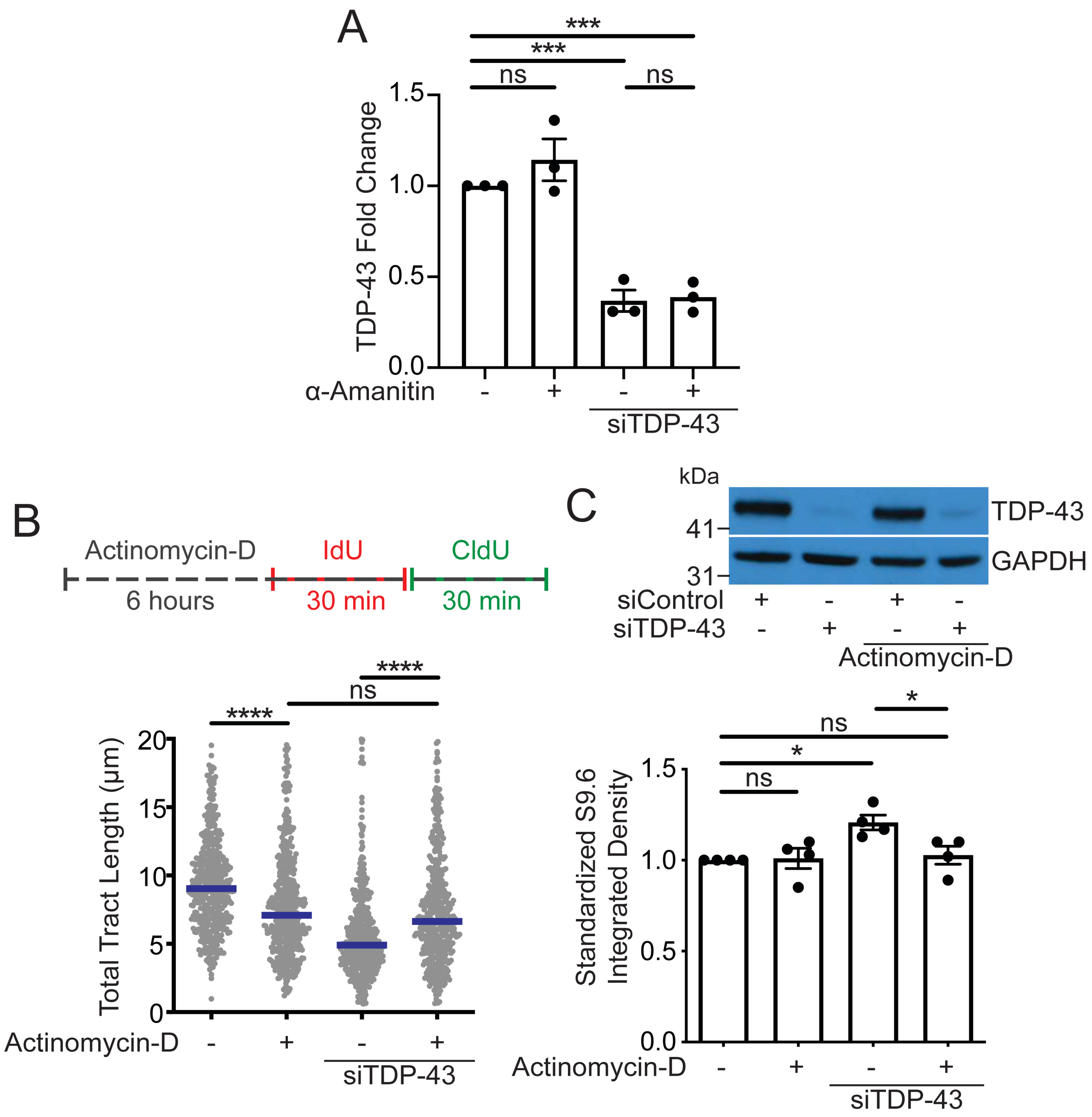

Figure S4: R-loop Resolution by Actinomycin-D Resolves Replication Stress. (A) Quantification of TDP-43 protein expression after α-amanitin treatment in HeLa cells (mean ± SEM) from three independent experiments. Statistics: paired t-test; ns, non-significant, ***$P \leq 0.0006$. (B) Top, schematic of the DNA fiber assay with the transcription inhibitor actinomycin-D. 1 μM actinomycin-D was added to cell culture media for 6 hours prior to DNA fiber assay and kept in the media throughout the labeling with IdU and CldU. Bottom, size distribution of total tract length (IdU + CldU) in HeLa cells after transfection with control siRNA or siTDP-43 and actinomycin-D treatments. Data are pooled from three independent experiments and shown as dot plot. Bars represent the median of $n \geq 150$ tracts scored for each data set. Statistics: Kruskal-Wallis test with Dunn's multiple comparisons test; ns, non-significant, ****$P < 0.0001$. (C) Top, TDP-43 protein levels after siRNA knockdown (siTDP-43) and actinomycin-D treatments detected by western blot. Bottom, quantification of S9.6 intensity signal shown as relative to control cells. Five separate data sets were normalized to respective negative controls. $n \geq 100$ cells scored for each data set. Data are represented as mean ± SEM. Statistics: RM one-way ANOVA with Tukey's multiple comparisons test, with a single pooled variance; ns, non-significant, *$P \leq 0.0253$.

Journal of Cell Science • Supplementary information

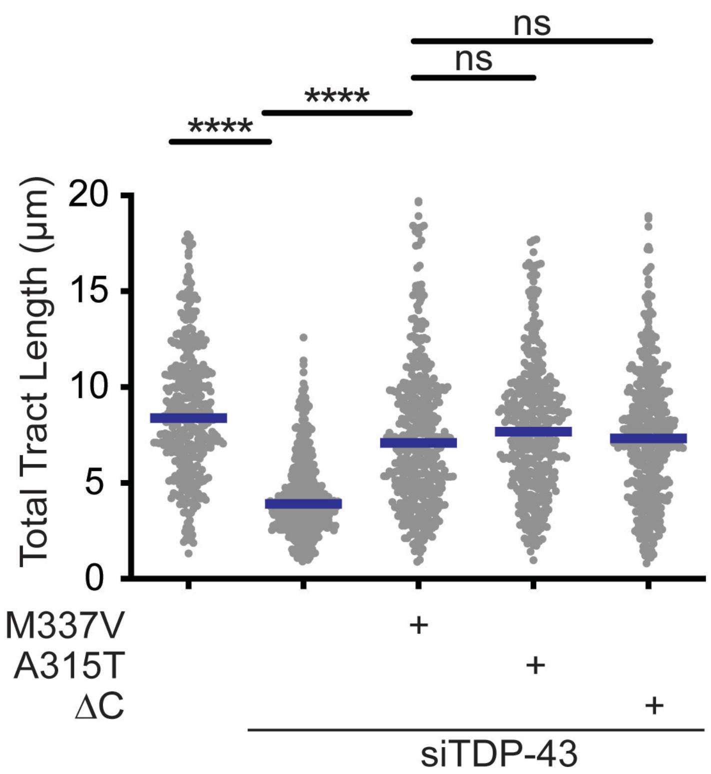

Figure S5: TDP-43 C-terminal Mutations Do Not Alter DNA Replication. Size distribution of total tract length (IdU + CldU) in HeLa cells after transfection with control siRNA or siTDP-43 and transfection with siRNA-resistant TDP-43 mutants M337 or A315T. M337V and A315T mutants are point mutations in the C-terminal domain of TDP-43 that represent common familial TDP-43 mutations. Data are pooled from three independent experiments. Bars represent the median of $n \geq 150$ tracts scored for each data set. Statistics: Kruskal-Wallis test with Dunn's multiple comparisons test; $^{****}P < 0.0001$.

Journal of Cell Science • Supplementary information

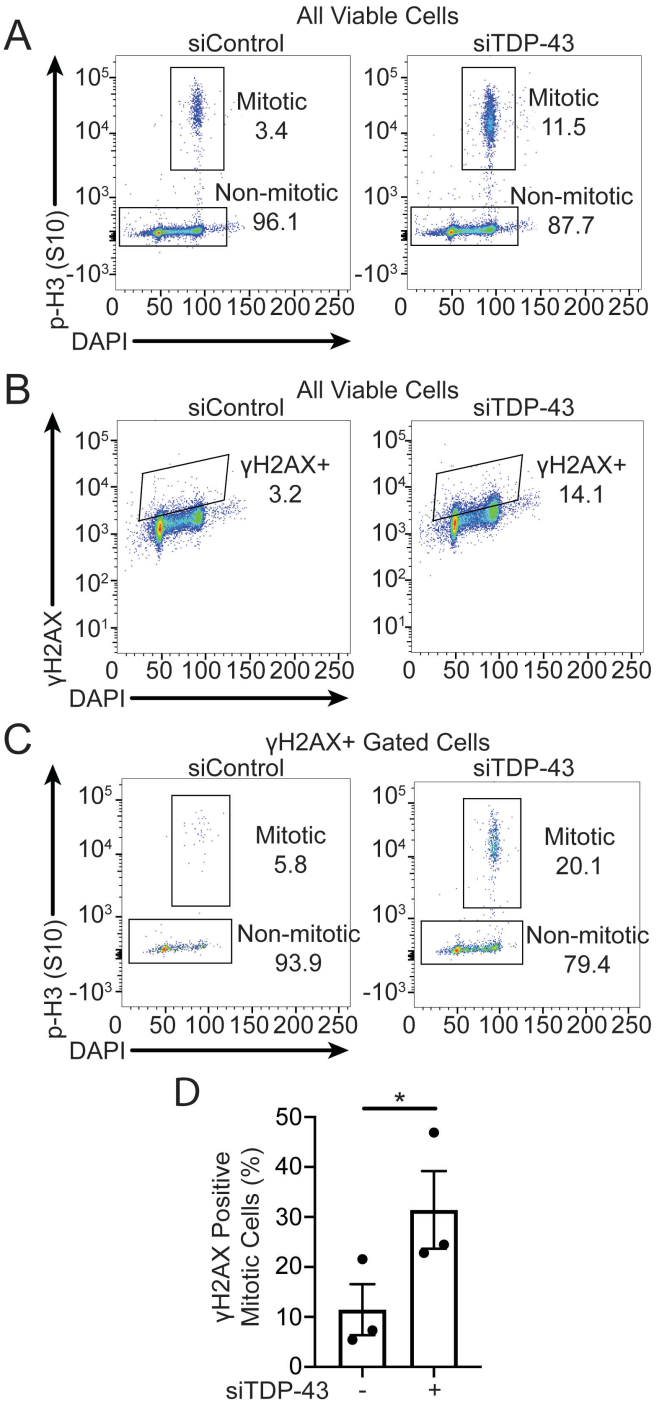

Figure S6: TDP-43 Loss Induces DNA Damage in Mitotic Cells. Cell cycle profiles detected by flow cytometry in siControl and siTDP-43 transfected HeLa cells. Cells were labeled with mitotic marker p-H3 (S10), and DNA damage marker γH2AX. (A) Plot showing p-H3 (S10) against DAPI of all viable cells to highlight mitotic and non-mitotic cells. (B) Plot showing γH2AX against DAPI of all viable cells to highlight DNA damage present. (C) Plot showing p-H3 (S10) against DAPI of only γH2AX+ cells to highlight DNA damage present in mitotic cells. (D) Quantification of γH2AX positive cells that are mitotic and stain positive for p-H3 (S10) (mean ± SEM) from 3 independent experiments. Statistics: paired t-test; *P = 0.0201.

Journal of Cell Science • Supplementary information

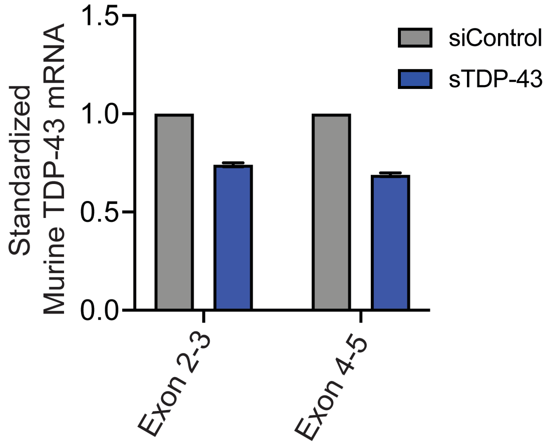

Figure S7: TDP-43 Downregulation in primary neurons. Real-time PCR quantifying TDP-43 mRNA from primary neurons treated with siTDP-43 or control. Specific oligonucleotides amplifying across exons 2-3 and 4-5 of the mouse *Tardbp* transcript were used. Mouse 36B4 was used as reference gene. Data are represented as mean ± SEM from three replicates.
